# Disrupted Calcium Release as a Mechanism for Atrial Alternans Associated with Human Atrial Fibrillation

**DOI:** 10.1371/journal.pcbi.1004011

**Published:** 2014-12-11

**Authors:** Kelly C. Chang, Jason D. Bayer, Natalia A. Trayanova

**Affiliations:** 1 Institute for Computational Medicine, Department of Biomedical Engineering, Johns Hopkins University, Baltimore, Maryland, United States of America; 2 IHU-LIRYC - L'Institut de RYthmologie et Modélisation Cardiaque, University of Bordeaux, Bordeaux, France; University of California San Diego, United States of America

## Abstract

Atrial fibrillation (AF) is the most common cardiac arrhythmia, but our knowledge of the arrhythmogenic substrate is incomplete. Alternans, the beat-to-beat alternation in the shape of cardiac electrical signals, typically occurs at fast heart rates and leads to arrhythmia. However, atrial alternans have been observed at slower pacing rates in AF patients than in controls, suggesting that increased vulnerability to arrhythmia in AF patients may be due to the proarrythmic influence of alternans at these slower rates. As such, alternans may present a useful therapeutic target for the treatment and prevention of AF, but the mechanism underlying alternans occurrence in AF patients at heart rates near rest is unknown. The goal of this study was to determine how cellular changes that occur in human AF affect the appearance of alternans at heart rates near rest. To achieve this, we developed a computational model of human atrial tissue incorporating electrophysiological remodeling associated with chronic AF (cAF) and performed parameter sensitivity analysis of ionic model parameters to determine which cellular changes led to alternans. Of the 20 parameters tested, only decreasing the ryanodine receptor (RyR) inactivation rate constant (ki_Ca_) produced action potential duration (APD) alternans seen clinically at slower pacing rates. Using single-cell clamps of voltage, fluxes, and state variables, we determined that alternans onset was Ca^2+^-driven rather than voltage-driven and occurred as a result of decreased RyR inactivation which led to increased steepness of the sarcoplasmic reticulum (SR) Ca^2+^ release slope. Iterated map analysis revealed that because SR Ca^2+^ uptake efficiency was much higher in control atrial cells than in cAF cells, drastic reductions in ki_Ca_ were required to produce alternans at comparable pacing rates in control atrial cells. These findings suggest that RyR kinetics may play a critical role in altered Ca^2+^ homeostasis which drives proarrhythmic APD alternans in patients with AF.

## Author Summary

Atrial fibrillation is an irregular heart rhythm affecting millions of people worldwide. Effective treatment of this cardiac disorder relies upon our detailed knowledge and understanding of the mechanisms that lead to arrhythmia. Recent clinical observations have suggested that alternans, a phenomenon where the shape of the electrical signal in the heart alternates from beat to beat, may play an important role in this process, but the underlying mechanisms remain unknown. In this study, we use computational models to conduct a detailed examination of the causes and contributors to alternans associated with human atrial fibrillation. We find that in atria remodeled by atrial fibrillation, alternans appears near resting heart rates because several aspects of calcium cycling are disrupted in the atrial cells. In particular, the release and uptake of calcium from the cellular storage compartment, the sarcoplasmic reticulum, becomes imbalanced, leading to alternation in calcium signals from beat to beat. These findings provide important insights into the mechanisms of proarrhythmic alternans in human atrial fibrillation which may be used to develop novel therapeutic targets and treatment strategies in the future.

## Introduction

Atrial fibrillation (AF) is currently the most common cardiac rhythm disorder, posing a significant medical and economic challenge for the US health care system [1], [2]. This burden is likely to increase as the population ages and AF prevalence rises [3]. Effective prevention and treatment of AF depends upon advances in our understanding of underlying disease mechanisms. Although several features of AF electrophysiological remodeling have been identified over the past decades [4], [5], our knowledge about the arrhythmogenic substrate remains incomplete.

Beat-to-beat alternation in the shape of cardiac electrical signals, a phenomenon called alternans, has been observed in the atria of AF patients, but the mechanism underlying these alternans is not known [6]–[11]. Narayan *et al.* reported differences in the rate dependence of action potential duration (APD) alternans in patients, with APD alternans occurring at pacing rates near rest in AF patients but only at fast pacing rates in controls [8]. Narayan *et al.* also found that APD alternans always preceded AF initiation, indicating that alternans may play an important role in establishing the arrhythmogenic substrate and creating vulnerability to AF. Thus, a better understanding of AF arrhythmogenesis will likely depend upon identification of the mechanism driving atrial alternans at heart rates near rest.

Interestingly, in AF patients the slope of the APD restitution curve was <1 during APD alternans onset at slow pacing rates. This suggests that a cellular mechanism other than voltage-driven instability underlies APD alternans at heart rates near rest [9]. Altered Ca^2+^ handling in atrial myocytes is known to play a crucial role in the generation of AF triggers and in AF maintenance [12], [13]. Ca^2+^ cycling instabilities have been shown to underlie ventricular alternans in heart failure [14], [15], as well as atrial alternans in several non-AF animal models [16]–[18]. However, it is unknown whether these represent a plausible mechanism for atrial alternans in AF patients, particularly at heart rates near rest. We therefore sought to determine, using a computer model of human atrial tissue, whether Ca^2+^ handling abnormalities, or other electrophysiological changes that occur in AF, lead to APD alternans. We identified a critical change in the kinetics of the ryanodine receptor (RyR) that was responsible for APD alternans onset at slower pacing rates, and subsequently aimed to elucidate the mechanistic relationship between this disruption in RyR kinetics and alternans onset. To this end, we employed single-cell clamping of ionic model parameters and iterated map analysis in order to dissect the mechanisms which drive alternans in atrial tissue, as well as to provide important insights into the pathophysiological changes that contribute to the development of alternans in AF patients.

## Results

### APD alternans in the human AF tissue model

**Figure 1:**
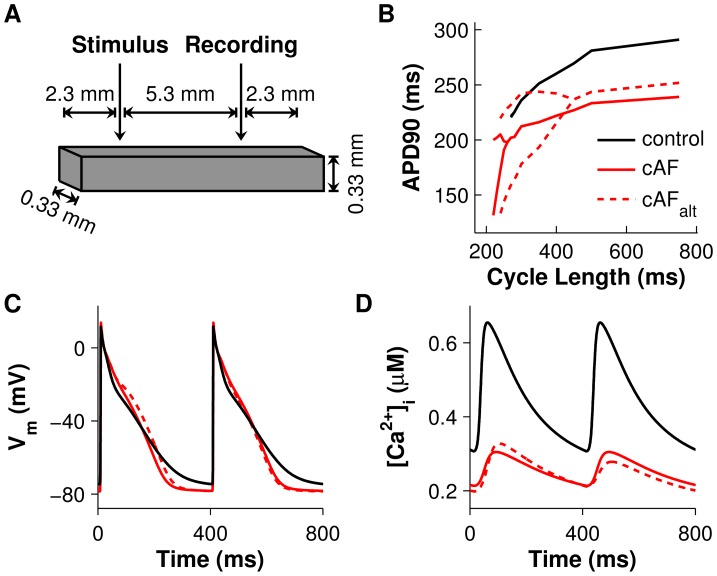
Tissue preparation setup and comparisons of control, cAF, and cAF_alt_ tissue during pacing. (A) Atrial tissue mesh with stimulus and recording electrodes. (B) APD restitution curves for control tissue (black), cAF-remodeled tissue [19] (red), and cAF_alt_ tissue with APD alternans onset and amplitude matching clinical data [8] (dotted red line). The RyR inactivation rate constant (ki_Ca_) was reduced 50% in the cAF model to create the cAF_alt_ model. APs (C) and CaTs (D) recorded from the last two beats at 400-ms pacing CL. Alternans are present in the cAF_alt_ tissue but not in control or cAF tissue.

In order to investigate ionic mechanisms in human AF that contribute to the generation of atrial APD alternans at the tissue level, we created a computer model of human atrial tissue incorporating ionic remodeling associated with chronic AF (cAF), as described in Methods. The sensitivity of APD alternans to ionic model parameters was evaluated by varying parameters one at a time and applying the clinical pacing protocol used by Narayan *et al.* to induce APD alternans in AF patients [8] (see Table 1 and Methods). For control, a model of normal human atrial tissue was also simulated. We then assessed the magnitude and onset pacing cycle length (CL) of APD alternans by analyzing voltage traces from the recording electrode (Fig. 1A), as outlined in Methods.

**Table 1:** Ionic model parameters used in parameter sensitivity analysis.

Parameter	Description
g_Na_	Maximal fast Na^+^ current conductance
g_NaL_	Maximal late Na^+^ current conductance
g_CaL_	Maximal L-type Ca^2+^ current conductance
τ_f_	L-type Ca^2+^ current voltage-dependent inactivation time constant
τ_fCa_	Maximal L-type Ca^2+^ current calcium-dependent inactivation time constant
g_K1_	Maximal inward rectifier K^+^ current conductance
g_Kr_	Maximal rapidly activating delayed rectifier K^+^ current conductance
g_Ks_	Maximal slowly activating delayed rectifier K^+^ current conductance
g_Kur_	Maximal ultrarapid delayed rectifier K^+^ current conductance
g_to_	Maximal transient outward K^+^ current conductance
Ibar_NCX_	Maximal Na^+^/Ca^2+^ exchanger current
k_s_	SR Ca^2+^ release rate constant
ki_m_	Transition rate constant for the RyR
ko_m_	Transition rate constant for the RyR
ki_Ca_	Baseline inactivation rate constant for the RyR without luminal SR Ca^2+^ dependence
ko_Ca_	Baseline activation rate constant for the RyR without luminal SR Ca^2+^ dependence
k_leak_	SR Ca^2+^ leak rate constant
V_maxSRCaP_	V_max_ of SERCA pump
ec_50SR_	EC_50_ for luminal Ca^2+^ dependence of the RyR
K_mf_	K_m_ for SERCA pump in forward mode

**Figure 2:**
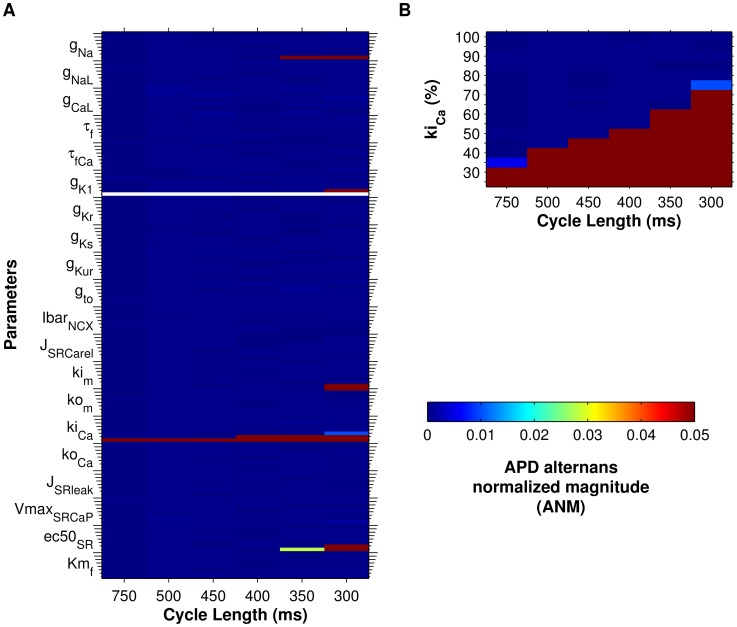
Sensitivity of APD alternans magnitude to ionic model parameters in cAF tissue. Parameter sensitivity analysis was performed in cAF tissue in order to identify ionic model parameters that influence alternans. For panels A and B, APD alternans normalized magnitude (ANM) is indicated by the colorbar (>0.05 considered significant). (A) Parameters were scaled one at a time between 25% (short ticks) and 200% (long ticks) of their AF model values (25% increments). Only decreasing the RyR inactivation rate constant (ki_Ca_) produced alternans at the longest CLs. (B) ki_Ca_ was scaled between 25% and 100% in 5% increments, producing a range of APD alternans onset CLs between 300–750 ms.

In the control model, significant APD alternans did not occur before loss of capture at 260 ms CL (Fig. 1B). However, in the cAF-remodeled tissue preparation, significant APD alternans appeared at a CL of 240 ms (Fig. 1B). Varying the RyR inactivation rate constant (ki_Ca_) had the greatest effect on alternans onset CL in the human cAF-remodeled tissue (Fig. 2A). In fact, only reduction of ki_Ca_ resulted in alternans onset at CLs of 300–500 ms (Fig. 2B), matching alternans onset CLs observed in AF patients [8]. When other ionic model parameters were varied from their original cAF values, APD alternans either did not appear in the tissue model at CL≥300 ms (Fig. 2A, blue areas), appeared only at CL≤350 ms (Fig. 2A, red areas), or did not appear before loss of capture or conduction block occurred in the tissue (Fig. 2A, white spaces). These results suggest that altered RyR kinetics is the critical cellular component underlying the occurrence of APD alternans in AF patients at pacing rates near rest, and that ki_Ca_ plays a key role in this process.

We also tested whether differences between left and right atrial electrophysiology affect alternans susceptibility using a right atrium (RA) version of the cAF model [19] in tissue simulations. Results for RA tissue were very similar to those for the left atrium (LA), demonstrating that modulation of ki_Ca_ could reproduce alternans observed at pacing rates near rest in both the LA and RA of AF patients [8] (S2 Figure).

**Figure 3:**
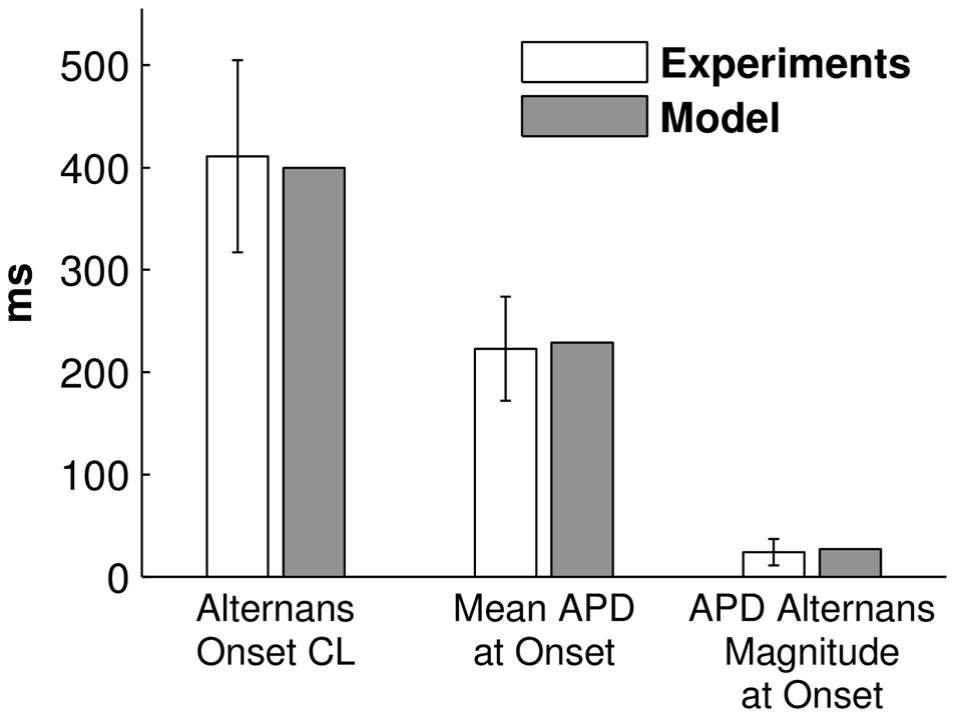
Comparison of alternans onset characteristics in persistent AF patients and in the cAF_alt_ tissue model. Mean±SD alternans onset data during pacing in persistent AF patients (white bars) was taken from Table 2 in Ref. [8]. When the cAF_alt_ tissue model was paced similarly, alternans onset CL, mean APD at onset, and APD alternans magnitude at onset were within one SD of clinical data (gray bars).

**Figure 4:**
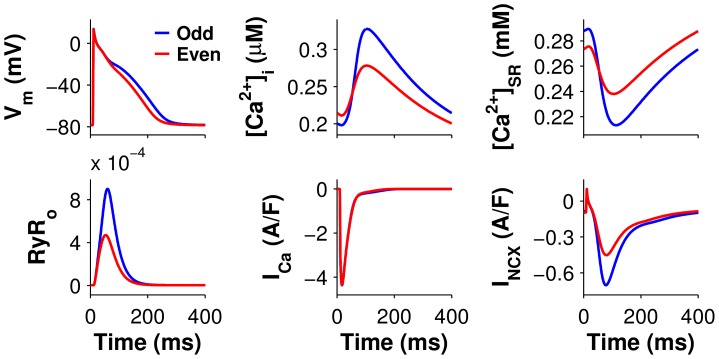
Alternans in cAF_alt_ tissue at the onset CL. The odd (blue) and even (red) beats at the alternans onset CL (400 ms) are shown superimposed. Large Ca^2+^ release occurred during the long beat (blue traces). Top (left to right): transmembrane potential (V_m_), intracellular Ca^2+^ ([Ca^2+^]_i_), and SR Ca^2+^concentration ([Ca^2+^]_SR_). Bottom (left to right): RyR open probability (RyR_o_), L-type Ca^2+^ current (I_Ca_), Na^+^/Ca^2+^ exchanger current (I_NCX_).

When ki_Ca_ was decreased by 50% in the cAF model (we refer to this as the cAF_alt_ ionic model), APD alternans onset data from the human AF tissue model agreed well with data from persistent AF patients. Significant APD alternans began at 400-ms CL (Fig. 1B, dotted red line), mean APD at onset was 229 ms, and APD alternans magnitude at onset was 27 ms (Fig. 1C, dotted red line). These metrics were each within one standard deviation (SD) of clinical observations [8] (Fig. 3). The cAF_alt_ model also displayed noticeable alternans in intracellular Ca^2+^ ([Ca^2+^]_i_) at the onset CL (Fig. 1D). For both the cAF and cAF_alt_ models, mean APDs were shorter than in the control model (Fig. 1B–C), and diastolic and systolic [Ca^2+^]_i_ were lower than in control (Fig. 1D). At 400-ms CL in the cAF_alt_ model, on the odd (long) vs. the even (short) beat (Fig. 4, blue vs. red), there was higher sarcoplasmic reticulum (SR) Ca^2+^ load before release (0.288 vs. 0.273 mM), higher peak RyR open probability (RyR_o_) (9.0e-4 vs. 4.7e-4), a larger intracellular Ca^2+^ transient (CaT) amplitude (Δ[Ca^2+^]_i_ = 0.13 vs. 0.067 µM), similar L-type Ca^2+^ (LCC) current (integrated over one beat: 144 vs. 140 mC/F), and increased Na^+^/Ca^2+^ exchanger (NCX) current (I_NCX_, integrated over one beat: 98.4 vs. 74.5 mC/F). The positive coupling between transmembrane potential (V_m_) and Ca^2+^, with I_NCX_ as the primary electrogenic current, is consistent with experimental findings [20]. Since the magnitude and onset of APD alternans in the cAF_alt_ model provided the best agreement with clinical APD alternans data (Fig. 3), we chose to use this model for subsequent investigations into the underlying causes of alternans occurrence.

### SR Ca^2+^ release underlies alternans onset

**Figure 5:**
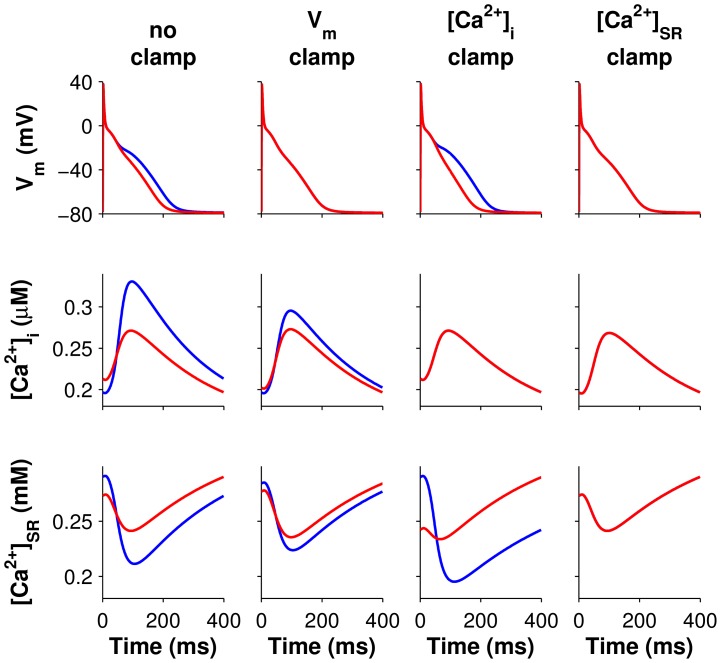
Voltage and Ca^2+^ even beat clamps for the single-cell cAF_alt_ model. Traces of transmembrane potential (V_m_, row 1), intracellular Ca^2+^ ([Ca^2+^]_i_, row 2), and SR Ca^2+^ ([Ca^2+^]_SR_, row 3) from two consecutive beats are superimposed to show alternans between even (red) and odd (blue) beats. Column 1: the unclamped cAF_alt_ cell paced to steady state at 400-ms CL displayed alternans in V_m_ and Ca^2+^. The red traces depicted in column 1 were used to clamp V_m_ (column 2), [Ca^2+^]_i_ (column 3), or [Ca^2+^]_SR_ (column 4). Alternans persisted when V_m_ or [Ca^2+^]_i_ is clamped, but clamping [Ca^2+^]_SR_ eliminated alternans.

**Figure 6:**
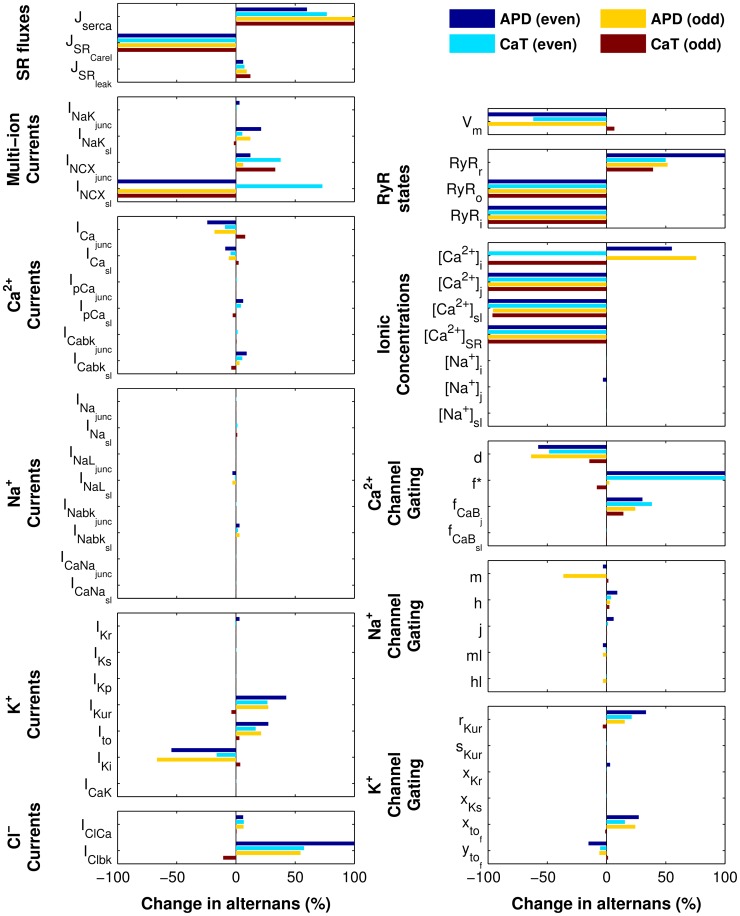
Summary of ionic model variable clamps for the single-cell cAF_alt_ model. Results for all ionic model variable clamping simulations are summarized in bar graphs showing the percent changes in APD and CaT alternans magnitudes when model variables were clamped to even or odd beat waveforms. Alternans were eliminated (>99% decrease in APD and CaT alternans magnitudes for both even and odd beat waveforms) only when SR release variables were clamped (SR Ca^2+^ release flux, J_SRCarel_; RyR open probability, RyR_o_; RyR inactivated probability, RyR_i_; SR Ca^2+^ ([Ca^2+^]_SR_); and junctional Ca^2+^([Ca^2+^]_j_). Gating variable f (asterisk) displayed higher order instability when clamping to the even beat waveform, so the increase in alternans magnitude was considered infinitely large. Left column: SR fluxes and sarcolemmal currents. Right column: state variables.

Since APD alternans throughout the homogenous cAF_alt_ tissue preparation were concordant and of similar magnitude (S3 Figure), electrotonic effects and CV restitution were excluded as factors influencing these alternans. Indeed, APD and CaT alternans in the cAF_alt_ tissue model were very similar to alternans in the isolated single-cell cAF_alt_ model (Fig. 5, left column vs. Fig. 4, top row). We therefore concluded that cellular mechanisms gave rise to alternans in the cAF_alt_ tissue model and decided to utilize single-cell simulations in order to investigate these mechanisms. We first used the ionic model variable clamping protocol described in detail in Methods. The percent change in APD and CaT alternans magnitudes, when each ionic model variable was clamped to its trace from either the even (short) or odd (long) steady-state beat at the alternans onset CL (400 ms), are summarized in Fig. 6 (right column: state variables, left column: currents and fluxes). Variables which resulted in >99% reduction in APD and CaT alternans magnitudes for both even and odd beat clamps were considered essential for alternans.

Clamping V_m_ resulted in −61.8% change in CaT alternans magnitude for even beat clamps and +6.6% for odd beat clamps, demonstrating that the alternans were not voltage-driven (see even and odd beat clamps depicted in column 2 of Fig. 5 and S4 Figure, respectively). Clamping [Ca^2+^]_i_ enhanced APD alternans (+55.2% and +75.8% for even and odd beat clamps, respectively, column 3 of Fig. 5 and S4 Figure). However, when SR Ca^2+^ ([Ca^2+^]_SR_) was clamped to either the even or odd beat waveforms, alternans in both APD and CaT were eliminated (<−99%), demonstrating that the alternans were driven by SR Ca^2+^ instability (column 4 of Fig. 5 and S4 Figure). In addition, four other variables could be clamped to the even or odd beat waveforms to eliminate APD and CaT alternans: RyR inactivated probability (RyR_i_), RyR open probability (RyR_o_), junctional Ca^2+^ ([Ca^2+^]_j_), and SR Ca^2+^ release flux (J_SRCarel_) (Fig. 6, and S5 and S6 Figures). All five of these variables were therefore critical for enabling alternans to occur at the onset CL. Furthermore, these variables directly impact SR Ca^2+^ release, implicating SR Ca^2+^ release as the underlying source of alternans in the cAF_alt_ model.

There were two ionic model components which greatly reduced but did not eliminate alternans when clamped: sub-sarcolemmal Ca^2+^ ([Ca^2+^]_sl_) and sub-sarcolemmal Na^+^/Ca^2+^ exchanger current (I_NCXsl_). Clamping [Ca^2+^]_sl_ to the even beat eliminated all alternans; clamping to the odd beat greatly reduced APD and CaT alternans (−95.8% and −96.2%, respectively), although large alternation in SR load persisted (Fig. 6 and columns 1–2 of S7 Figure). Similarly, clamping I_NCXsl_ to the even beat waveform resulted in elimination of APD but not CaT alternans (+72.9%), while clamping to the odd beat waveform resulted in elimination of all alternans (Fig. 6 and columns 3–4 of S7 Figure). Hence, the SR Ca^2+^-driven instabilities produced alternans in Ca^2+^ cycling which were positively coupled to voltage through I_NCXsl_ and [Ca^2+^]_sl_.

### Steepening of the SR Ca^2+^ release slope results in alternans

**Figure 7:**
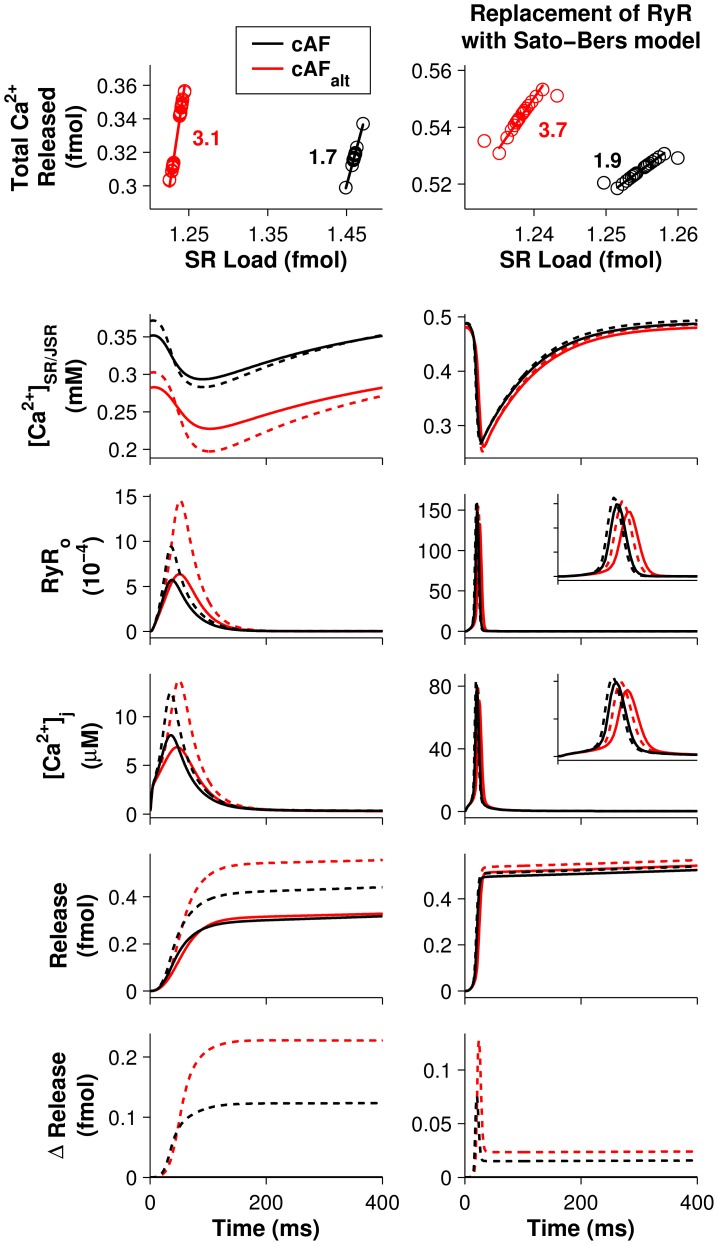
The effect of RyR inactivation on SR Ca^2+^ release slope. Left column: simulations using the original cAF (black) and cAF_alt_ (red) models. Right column: simulations in which the original RyR model, which included Ca^2+^-dependent inactivation, was replaced with the Sato-Bers RyR model, which utilizes calsequestrin regulation instead (see Table 2). In the Sato-Bers model, the SR is divided into junctional (JSR) and network (NSR) compartments. Top row: Total Ca^2+^ released from the SR is plotted against SR Ca^2+^ load under AP voltage clamp conditions (CL = 400 ms). The line of best fit is also plotted, with its slope value (the SR Ca^2+^ release slope) shown next to the data points. (In column 2, the first beat was excluded.) Modulating RyR inactivation by reducing ki_Ca_ (left column) or k_34_ (right column) by 50% increased the SR Ca^2+^ release slope in both models. Rows 2–6: Traces from a similar set of AP voltage clamp simulations. After reaching steady state (solid lines), SR or NSR load was perturbed at the beginning of the beat by a large amount (+20 µM, dashed lines) to illustrate the changes affecting SR Ca^2+^ release slope. Row 2: SR (JSR) Ca^2+^ ([Ca^2+^]_SR/JSR_). Row 3: RyR open probability (RyR_o_). Row 4: junctional Ca^2+^ ([Ca^2+^]_j_). Row 5: total Ca^2+^ released. Row 6: the difference in total Ca^2+^ release between perturbed and unperturbed (steady state) simulations. Insets in column 2, rows 3–4 show traces from t = 0–50 ms.

Increased steepness of the SR release-load relationship is a well-known mechanism for CaT alternans [21], [22]. The importance of SR Ca^2+^ release variables for APD and CaT alternans, as demonstrated by the results in Fig. 5, 6, and S4, S5, S6 Figures, led us to hypothesize that such a mechanism might give rise to Ca^2+^-driven alternans in the cAF_alt_ model at pacing rates near rest. To test this, we compared the cAF and cAF_alt_ ionic models under action potential (AP) voltage clamp conditions so that changes in CaT alternans would be due solely to changes in Ca^2+^ homeostasis rather than bidirectional coupling between V_m_ and Ca^2+^. After clamping each ionic model at a CL of 400 ms until steady state was reached, we perturbed [Ca^2+^]_SR_ and tracked SR load and SR Ca^2+^ release on the subsequent clamped beats (see Methods for details). The SR release-load relationships for the cAF (black) and cAF_alt_ (red) ionic models are depicted in Fig. 7 (left column, row 1). The slope of the release-load relationship in the cAF_alt_ model (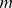 = 3.1) was much greater than the slope in the cAF model (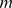 = 1.7), confirming our hypothesis that differences between the cAF and cAF_alt_ ionic models led to a steepening of the SR Ca^2+^ release slope.

To better explain the differences between the cAF and cAF_alt_ ionic models that gave rise to different SR Ca^2+^ release slopes, we first compared [Ca^2+^]_SR_, RyR_o_, [Ca^2+^]_j_, and cumulative Ca^2+^ release for the two models at steady state (Fig. 7, left column, rows 2–5, solid lines). In the cAF_alt_ model, [Ca^2+^]_SR_ at steady state was 19.7% lower than in the cAF model as a result of increased RyR opening (Fig. 7, left column, rows 2 and 3, red vs. black solid lines). Although this led to a 15.2% decrease in peak [Ca^2+^]_j_ in the cAF_alt_ model, the duration of the release event was prolonged (Fig. 7, left column, row 4, red vs. black solid lines). Consequently, though cumulative Ca^2+^ release in the cAF_alt_ model initially lagged behind, at t≈90 ms it actually surpassed the cumulative release in the cAF model, ultimately resulting in a 3.4% increase in total release by the end of the beat (Fig. 7, left column, row 5, red vs. black solid lines).

To illustrate how these differences between the cAF and cAF_alt_ ionic models impacted SR release slope, we applied a large perturbation to [Ca^2+^]_SR_ (+20 µM) at the beginning of a clamped beat and compared the unperturbed (steady state, solid line) and perturbed (dotted line) traces for each model (Fig. 7, left column, rows 2–6). Higher SR load at the beginning of the beat led to increased SR release flux due to luminal Ca^2+^ regulation of the RyR (causing more opening), as well as to the increased concentration gradient between the SR and junctional compartments. In both the cAF and cAF_alt_ models, these changes led to increased peak [Ca^2+^]_j_ (+54.4% and +100%, respectively) and RyR opening (+64.6% and +129%, respectively) as a result of more Ca^2+^-induced Ca^2+^ release (Fig. 7, left column, rows 2–4). The positive feedback relationship between [Ca^2+^]_j_ and RyR opening was strong enough such that when SR load was increased (Fig. 7, left column, row 2, dotted vs. solid lines), this actually resulted in a lower minimum [Ca^2+^]_SR_ during release (−3.6% and −13.3% for cAF and cAF_alt_ models, respectively). However, the amount of positive feedback differed between the cAF and cAF_alt_ ionic models. Positive feedback amplifies changes in release inputs, such as SR load; therefore, in the cAF model, where [Ca^2+^]_j_ is higher and positive feedback is stronger, the increase in [Ca^2+^]_SR_ produced a slightly greater change in release (compared to the unperturbed, steady state simulation) during the rising phase of [Ca^2+^]_j_ (t<48 ms) than in the cAF_alt_ model (Fig. 7, left column, row 6, black vs. red).

By contrast, termination of release occurs through a negative feedback process, with RyRs inactivating upon the binding of junctional Ca^2+^. Negative feedback attenuates changes in release so that robust, fast termination of release is achieved even when a disturbance (such as a transient increase in SR load) occurs. In the cAF_alt_ model, negative feedback is decreased both directly, via reduction of ki_Ca_, and indirectly, via reduction in [Ca^2+^]_j_ that occurs as a result of decreased SR load. This causes prolongation of the Ca^2+^ release event and a larger peak [Ca^2+^]_j_ (Fig. 7, left column, row 4, red vs. black dotted lines). Consequently, when SR load was increased by the same amount in the cAF and cAF_alt_ models, although the cAF_alt_ model had a lesser initial change in release because of weaker positive feedback, it also had a greater final change in release, i.e. a steeper SR release-load relationship, because of weaker negative feedback (Fig. 7, left column, row 6, red vs. black).

The results in column 1 of Fig. 7 demonstrate how the steeper SR release slope in the cAF_alt_ ionic model (as compared to the cAF ionic model) depends upon RyR inactivation by junctional Ca^2+^. However, recent work suggests that termination of release does not rely on direct Ca^2+^-dependent inactivation of the RyR but rather on local SR Ca^2+^ depletion [23]–[26]. In order to test whether steepening of the SR release slope could occur in the cAF model by an alternative release termination mechanism, we implemented a version of the cAF model in which the RyR Markov model was replaced with that of Sato and Bers and the SR was divided into junctional (JSR) and network (NSR) compartments [27] (see Table 2 and S1 Text). Termination of release in this alternative RyR model relies on calsequestrin (CSQN) binding to the RyR, which occurs as luminal [Ca^2+^] decreases causing changes in RyR opening and closing rates.

**Table 2:** RyR and SR parameters.

Parameters	Original cAF value [19]	Value used in replacement of RyR with Sato-Bers model [27]	Description
ko_Ca_	30 mM^−2^ ms^−1^	N/A	RyR opening rate
K_u_	N/A	15 ms^−1^	CSQN-unbound RyR opening rate
K_b_	N/A	0.015 ms^−1^	CSQN-bound RyR opening rate
τ_b_	N/A	0.164 ms	CSQN binding time constant
τ_u_	N/A	312 ms	CSQN unbinding time constant
τ_tr_	N/A	5 ms	JSR refilling time constant
V_SR_/V_cell_	0.035	N/A	SR fractional volume
V_JSR_/V_cell_	N/A	0.0035	JSR fractional volume
V_NSR_/V_cell_	N/A	0.0315	NSR fractional volume
V_maxSRCaP_	5.31×10^−3^ mM/ms	5.04×10^−2^ mM/ms	V_max_ of SERCA pump
k_s_	25 ms^−1^	134 ms^−1^	SR Ca^2+^ release rate constant
B_max_csqn_	2.6 mM	0.4 mM	CSQN concentration
K_C_	0.65 mM	0.6 mM	Ca^2+^/CSQN dissociation constant

The effects of decreased RyR termination in the Sato-Bers RyR model are shown in the right column of Fig. 7. When the CSQN-bound RyR closing rate k_34_ (analagous to the inactivation rate ki_Ca_ in the original model) is decreased from 100% to 50% (cAF_alt_), steady-state Ca^2+^ concentrations change modestly as compared to the original RyR formulation (Fig. 7, black vs. red solid lines), but nevertheless display similar trends: [Ca^2+^]_JSR_ decreases by 1.5% (vs. 19.7%, row 2), peak [Ca^2+^]_j_ is reduced by 10.5% (vs. 15.2%, row 4) and delayed, and total release increases by 3.6% (vs. 3.4%, row 5). When [Ca^2+^]_NSR_ is perturbed in the Sato-Bers models by +20 µM, Ca^2+^ release increases more in the cAF_alt_ model than in the cAF model (Fig. 7, right column, row 6, red vs. black dotted lines). Consequently, the SR Ca^2+^ release slope is steeper in the cAF_alt_ model (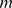 = 3.7 vs 1.9, Fig. 7, right column, row 1). Thus, although changes in SR Ca^2+^ release slope in the original cAF model are caused by altered junctional Ca^2+^-dependent inactivation, altered SR Ca^2+^-dependent mechanisms of release termination can produce such changes in SR Ca^2+^ release slope as well.

### Iterated map analysis

Although SR Ca^2+^ release slope is an important component of Ca^2+^ homeostasis, other aspects of Ca^2+^ cycling, such as SR Ca^2+^ uptake, could also have a significant impact. In order to understand how both SR release and uptake contribute to CaT alternans onset at slow pacing rates in human cAF cells, we used an iterated map analysis for investigating Ca^2+^ cycling stability under AP voltage clamp conditions. Three factors affecting Ca^2+^ cycling stability were included in the analysis: SR release, SR uptake, and cellular Ca^2+^ flux across the sarcolemma. The latter factor was included because Ca^2+^ content in the human atrial cell model varied significantly enough to affect alternans threshold predictions.

**Figure 8:**
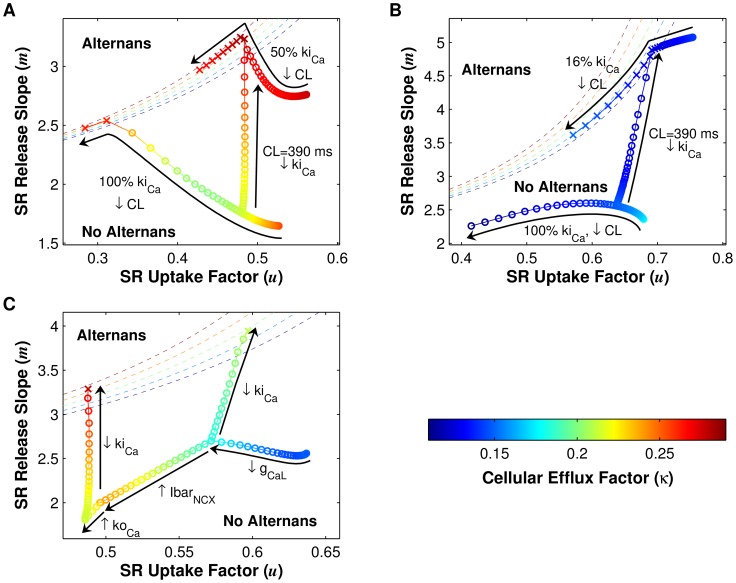
Iterated map analysis of Ca^2+^ cycling in cAF and control cells. For each panel, SR Ca^2+^ release slope (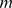) is plotted against SR Ca^2+^ uptake factor (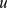), with cellular Ca^2+^ efflux factor (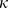) values indicated in the color bar. The boundaries between stable (no alternans) and unstable (alternans) regions in the 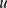-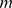 plane are denoted by dashed lines for different values of 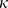 (see Eq. 1). Circles and X's indicate the absence and presence of alternans, respectively. (A) Results for the cAF model. CL is varied, from 700 ms to 200 ms for the 100% ki_Ca_ model and from 700 ms to 300 ms for the 50% ki_Ca_ model (i.e., the cAF_alt_ model), in 10-ms increments. At a CL of 390 ms, ki_Ca_ is scaled from 100% to 50% in 2% increments. (B) Same as in panel A, except that the control cell model is used, and ki_Ca_ is scaled from 100% to 16%. (C) Starting with the control cell parameter values, L-type Ca^2+^ current conductance (g_CaL_), maximal Na^+^/Ca^2+^ exchanger current (Ibar_NCX_), and RyR activation rate constant (ko_Ca_) are sequentially scaled to cAF values, resulting in net decreases in 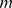 and 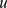. Finally, ki_Ca_ is scaled to 50% (as in the cAF_alt_ model), and 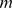 increases sufficiently to reach the alternans boundary (red X). If only g_CaL_ is decreased to the cAF value, then alternans threshold is achieved at a higher ki_Ca_ value (72%, green X).

For each version of the human atrial cell model (cAF and control), we calculated the SR Ca^2+^ release slope (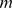), the SR Ca^2+^ uptake factor (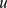), and the cellular Ca^2+^ efflux factor (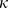) [28], [29] for a range of ki_Ca_ values and pacing rates and compared the value of 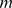 to the threshold for alternans. For a typical range of parameter values (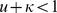, see S1 Text), the threshold value of 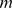 required for alternans is given by the following equation:
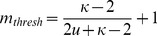
(1)Theoretical analysis predicts that the system is stable when 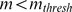. Eq. 1 is graphed for a range of 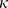 values in Figs. 8A–C (dotted lines). Each curve represents the boundary between stable (no alternans) and unstable (alternans) Ca^2+^ cycling in the 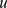-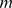 plane for a particular value of 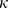. As 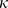 increases (Fig. 8A–C, dark blue to dark red), the threshold curve steepens, indicating that increased Ca^2+^ extrusion from the cell has a protective effect, helping to restore Ca^2+^ content back to steady state following a perturbation. Thus, a higher value of 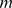 is required to reach alternans threshold for higher values of 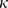. Note that in this theoretical approach, increased Ca^2+^ efflux (κ) has the opposite effect as in Qu *et al.* [29], suppressing rather than promoting Ca^2+^ alternans.

The effects of changing CL and changing ki_Ca_ are explored for the cAF model in Fig. 8A. At the default ki_Ca_ value (100%), as CL is decreased from 700 ms to 200 ms (−10 ms increments), 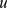 decreases, 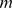 increases, and the system approaches the alternans threshold given by Eq. 1. The change in 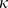 values is non-monotonic, initially decreasing (orange to green) and then increasing (green to orange) as CL is decreased. However, the change in 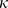 has a minimal effect at small 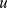 values, since the threshold curves for different 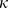 values converge at 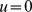. At CL<220 ms, the cell begins to display alternans in Ca^2+^ cycling, coinciding with the iterated map parameter values residing very close to the theoretically predicted boundary given by Eq. 1 (Fig. 8A, orange X's). When ki_Ca_ is set at 50% of the default cAF value (cAF_alt_ model), a similar trend is observed. However, the 50% ki_Ca_ cAF model reaches threshold at a lower pacing rate (CL = 390 ms for the 50% ki_Ca_ cAF model vs. 210 ms for the 100% ki_Ca_ cAF model, Fig. 8A, X's). This is primarily due to 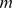 increasing as ki_Ca_ is decreased, illustrated by the trajectory of the system in the 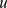-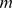 plane as CL is held constant at 390 ms but ki_Ca_ is decreased from 100% to 50% (Fig. 8A).

We next performed the same iterated map analysis for the control atrial cell model with varying CL and ki_Ca_ values (Fig. 8B). When ki_Ca_ is at 100%, 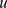 decreases as CL is decreased. However, unlike in the cAF model, in the control case the value of 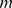 undergoes a net decrease as CL shortens from 700 to 200 ms. Ultimately, since both 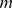 and 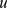 decrease as CL is shortened, the control atrial cell (with ki_Ca_ at 100%) fails to reach threshold and remains in the stable, no alternans region. This suggests that alternans in control patients, which occur at CL<250 ms [8], are driven by voltage rather than Ca^2+^. As in the cAF model, the alternans threshold CL in the control model can be adjusted by modulating the value of ki_Ca_ (Fig. 8B, CL = 390 ms). However, in the control model, ki_Ca_ must be decreased much more than in the cAF model in order to reach 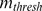 at a CL of 390 ms (ki_Ca_ reduced to 16% vs. 50%). The need for dramatic and possibly unrealistic reductions in ki_Ca_ to produce alternans at slow rates in control is consistent with the absence of alternans observed in control patients at CL≥250 ms [8].

To explain the difference in Ca^2+^ cycling properties of the cAF and control models, we examined the effects of cAF cellular remodeling on iterated map parameters. Stochastic ionic model parameter variation and regression analysis [30] (see S1 Text) predicted that of the ten model parameters altered in the control model to construct the cAF model, seven would have significant effects on alternans threshold CL (these are g_CaL_, g_Kur_, ko_Ca_, Ibar_NCX_, g_to_, g_K1_, and g_Na_, see S8 Figure). Of these seven parameters, three are involved in Ca^2+^ handling (g_CaL_, ko_Ca_, and Ibar_NCX_). The effects of changing these three parameters from control to cAF values is depicted sequentially in Fig. 8C: starting with the default values for the control cell at a CL of 390 ms, first g_CaL_ is decreased and then Ibar_NCX_ and ko_Ca_ are increased to cAF values, resulting in an overall decrease in 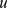 and 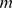. Finally, when ki_Ca_ is decreased to the cAF_alt_ value (50%), the large increase in 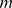 causes the system to reach 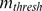 and alternate (Fig. 8C, red X). This illustrates why the control cell is less susceptible to CaT alternans than the cAF cell: at a given ki_Ca_ value and pacing rate, SR uptake efficiency (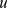) is higher in the control model, thus requiring a large increase in the pacing rate (which decreases 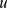) and/or a large decrease in ki_Ca_ (which increases 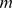) in order to reach 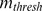. Of the three cAF parameters which decrease 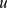, however, g_CaL_ is the most important for alternans onset, since remodeling of Ibar_NCX_ and ko_Ca_ decreases 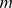, while remodeling of g_CaL_ increases 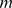. When g_CaL_ is remodeled and Ibar_NCX_ and ko_Ca_ remain at control values, only a 28% decrease in ki_Ca_ is required to reach 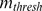 (Fig. 8C, green X).

## Discussion

### Findings and significance

The first goal of this study was to identify the electrophysiological changes in human atrial cells that are responsible for the occurrence of APD alternans at heart rates near rest, as observed in AF patients. Using parameter sensitivity analysis, we found that of the 20 electrophysiological model variables tested, only changes in the RyR inactivation rate constant (ki_Ca_) could produce APD alternans at relatively slow pacing rates in a tissue model of persistent/chronic AF. In particular, decreasing ki_Ca_ by 50% (the cAF_alt_ model) produced a good match to clinical data. We next aimed to provide mechanistic insight into why disruption of RyR kinetics, together with other electrophysiological changes occurring in AF, leads to alternans onset at pacing rates near rest. We established that alternans in the cAF_alt_ model at the onset CL were Ca^2+^-driven rather than voltage-driven, and that they depended upon SR Ca^2+^ release. Furthermore, CaT alternans occurred in the cAF_alt_ model at relatively long CLs because of steep SR Ca^2+^ release slope and decreased SR Ca^2+^ uptake efficiency. Lastly, we demonstrated that the ability to generate alternans at slower pacing rates by modulating ki_Ca_ depended upon the negative feedback properties of SR Ca^2+^ release.

This study is the first to identify a possible mechanism for alternans occurring at slow heart rates in AF patients. Our novel findings show that alternans at slow rates is Ca^2+^-driven, brought about by AF-associated remodeling of the Ca^2+^ handling system in atrial cells. Clinical and experimental research has shown that atrial alternans is associated with disease progression in AF patients [8] and with increased AF susceptibility after myocardial infarction [31], [32] and atrial tachycardia [33], [34] in animal models. Additionally, CaT alternans have been studied in animal atrial myocytes [17], [18], [35] and in the intact atria of AF-prone mice [36]. However, the precise cellular mechanism underlying alternans at heart rates near rest in the remodeled human atria has not been previously identified, and a direct relationship between human AF and CaT alternans in the atria has not been established until now. Elucidating the mechanism driving alternans at slow rates is particularly important because APD oscillations appear to be closely linked to AF initiation [8]. If APD alternans play a direct role in AF initiation, the onset of alternans at slower pacing rates would indicate an increased susceptibility to arrhythmia in AF patients, consistent with clinical observations [8]. Identification of this mechanism would thus provide a significant scientific and clinical benefit, improving our understanding of arrhythmogenesis and aiding in the development of new targeted therapies for AF.

In this study, we demonstrate how different aspects of AF remodeling contribute to Ca^2+^-driven alternans onset at slower heart rates using a theoretical analysis of Ca^2+^ cycling. This analysis allowed us to quantitatively assess CaT alternans threshold under AP voltage clamp conditions in a detailed electrophysiological model, providing valuable insights into the effects of AF electrophysiological remodeling on Ca^2+^ handling and alternans. Furthermore, we identify a critical aspect of SR Ca^2+^ release—inactivation of the RyR—which is necessary for CaT alternans to occur at slow heart rates. These findings extend mechanistic insight about proarrhythmic ventricular Ca^2+^ remodeling [15], [37], [38] to the atria and may inform new therapeutic strategies to target the RyR and suppress Ca^2+^-driven alternans in the atria for the purposes of preventing or treating AF [36], [39].

### RyR dysregulation in AF

The RyR has been the focus of several studies concerning trigger-mediated AF. In particular, disruption of RyR regulation has been shown to promote AF through increased RyR open probability, diastolic SR Ca^2+^ leak, and delayed afterdepolarizations [12], [39], [40]. Here we identify an additional pathological consequence of the disruption of RyR regulation in AF: Ca^2+^-driven alternans. Similar to what has been demonstrated with regards to Ca^2+^ sparks and triggered activity [39], we found that CaT alternans is coupled to voltage primarily through upregulated I_NCX_, thus driving the generation of APD alternans. The RyR's central role in both alternans and triggers has important clinical implications, given the proarrhythmic consequences of interaction between ectopic activity and the arrhythmogenic substrate created by voltage alternans [41]. New drug treatments to restore the normal function of the RyR and NCX, and thereby prevent arrhythmogenic triggers and alternans, have the potential to provide more effective alternatives to current AF drug therapies which target voltage-gated ion channels and often have proarrhythmic side effects [39].

The signaling pathways involved in RyR dysfunction in AF have been the focus of much active research over the past several years [39], [40]. Possible molecular mechanisms which could account for reduced RyR inactivation include RyR hyperphosphorylation by CAMKII and PKA and dissociation of the RyR subunit FKBP12.6, which have been shown to increase RyR open probability and promote arrhythmia [42], although the exact role of these mechanisms in RyR dysregulation are still debated [43]. Calmodulin has also been shown to interact directly with the RyR to decrease its open probability [44]. Metabolic factors may play a role, since modulation of the RyR as a result of glycolytic inhibition has been linked to atrial alternans in non-AF animal models [16], [17], [35]. Such metabolic impairment is thought to contribute to profibrillatory remodeling in the atria [45]–[47]. The cAF_alt_ model, with its reduction in ki_Ca_, can be considered a phenomenological representation of the various signaling pathway disruptions leading to alternans, which were not represented in the original cAF model. As more information becomes available, incorporation of these signaling mechanisms into computational models may provide additional insights into how reduction in RyR inactivation leads to Ca^2+^-driven alternans at slow heart rates in AF patients.

### The role of RyR refractoriness in CaT alternans

There is debate over whether CaT alternans depend primarily on SR Ca^2+^ load alternation or on RyR refractoriness [21], [41], [48]. Recent experiments [18], [49] and simulation studies [50]–[53] have shown that RyR refractoriness can drive CaT alternans under conditions where near-identical SR loads produce different amounts of SR release. In some simulation studies, this phenomenon was restricted to limited parameter values, clamping conditions, and cycle lengths [51], [52], while in a more recent modeling study focusing on atrial cells, SR load-independent alternans occurred over a broad range of pacing rates when the number of t-tubules was reduced [53]. Of note is the fact that many of these studies [51]–[53] utilized the same RyR gating scheme as this current study, yet they identified various mechanisms for CaT alternans. This demonstrates that the relative importance of the various mechanisms, whether SR load-driven, RyR refractoriness-driven, or otherwise, is highly context-dependent.

Although exploring the issue of SR load vs. RyR refractoriness was beyond the goals of the current study, our results suggest that in human cAF, both SR load alternation and RyR refractoriness are involved in alternans genesis at slower pacing rates. In our cAF_alt_ model, alternation in all SR Ca^2+^ release variables, including [Ca^2+^]_SR_, RyR open probability, and RyR inactivated probability, was necessary for alternans at the onset CL of 400 ms (Fig. 6). In addition, SR uptake flux (J_serca_) enhanced alternans when clamped (Fig. 6) and therefore suppressed alternans under normal pacing conditions, suggesting that SR load is indeed an important driver of CaT alternans in cAF and that upregulation of the SERCA pump may be an important therapeutic strategy for diminishing alternans. We also showed that CaT alternans occurred in the cAF_alt_ model at slow pacing rates because decreased RyR inactivation resulted in steepening of the SR release-load relationship. Together, these results indicate that the interplay between SR load and RyR kinetics is responsible for alternans onset in human AF.

### Other potential mechanisms for alternans susceptibility

The mechanisms for human atrial alternans susceptibility are likely to encompass a range of complex interactions at multiple scales of biology, which extend beyond the cellular-level mechanisms found here. In this study we examined the behavior of an atrial cell with well-developed t-tubules [19]. Research has shown that rat atrial cells have variable levels of t-tubule organization [54]. Such variation, if present in human atrial cells, would result in subcellular Ca^2+^ gradients which could make cells more susceptible to alternans [17], [55], [56]. Models of atrial myocytes incorporating detailed spatial descriptions [57] and local control of Ca^2+^ [58] will aid in future investigations of the subcellular mechanisms of cAF-related alternans.

In addition, the complex structure of the atria, including its normal conduction pathways [59] and fibrotic remodeling in AF [60], [61], may promote heterogeneity and discordant alternans, which significantly affect alternans dynamics and reentry initiation [9], [62]. Consideration of these factors in the future will further enrich the mechanistic insight gained from this current study and will advance our understanding of the role that alternans play in AF arrhythmogenesis.

### Limitations

In many cell models, the effective refractory period (ERP) is not consistent with ERP at the tissue level [63]. Electrotonic effects in tissue and the whole heart can shorten or lengthen APD depending on which structures and cell types are coupled to each other. Furthermore, alternans in single cell models may not be predictive of alternans in tissue, where conduction alternans can occur. This was the case for the control atrial tissue model, in which loss of capture occurred at a CL of 260 ms before reaching the very fast pacing rates at which APD alternans were observed in human control patients (CL = 218±30 ms) [8]. However, alternans onset at clinically observed rates occurred in the single-cell control model (200 ms CL, S9 Figure, black curve) and when ki_Ca_ was reduced by 5% (230 ms CL, S9 Figure, red curve). This suggests that the ionic model may not be well-constrained for tissue simulations at very fast rates. However, this issue did not affect the study of alternans onset at slower pacing rates, as was observed in AF patients.

Our ionic model variable clamping protocol, which involved separately clamping the even or odd beat waveforms, was used to test for model variables which could robustly suppress alternans when clamped to either of two very different waveforms. An alternative approach would be to clamp model variables to the single unstable, non-alternating waveform obtained using a control algorithm [64]. This approach would allow more precise assessment of fixed point stability, since clamping is done at the point of instability rather than during the bistable (alternans) endpoint. However, for the purposes of quantifying the most important variables influencing instability, the clamping protocol used in this study was sufficient to identify the central role of SR Ca^2+^ release, which was later confirmed through iterated map analysis.

Recent experimental evidence points towards local SR Ca^2+^ depletion, rather than Ca^2+^-dependent RyR inactivation, as the main mechanism of SR release termination [23]–[26]. Although alternans in the cAF_alt_ model relied on Ca^2+^-dependent RyR inactivation, other termination mechanisms which rely on SR Ca^2+^ (used in the Sato-Bers RyR model) may have similar effects on SR release slope and alternans susceptibility (Fig. 7, column 2). However, with the Sato-Bers RyR model, alternans and other complex oscillations began at the baseline pacing rate (750 ms CL, S10 Figure) and did not display the same rate dependence observed in patients [8]. In addition, large oscillations in CaT amplitude did not couple as strongly to voltage as with the original RyR, and oscillations were also attenuated in tissue (S10 Figure). Further work is needed to develop atrial cell models which incorporate current mechanistic understanding of SR Ca^2+^ release and which can also reproduce AF-related alternans rate dependence in tissue.

### Conclusion

AF is associated with progressive changes in alternans onset in the human atria, with alternans occurring at slower heart rates as AF severity worsens. We found that the differences in alternans onset between AF and control patients could be accounted for by changes in the inactivation rate of the RyR in a model of human atrial cAF-remodeled tissue. Single-cell simulations revealed that alternans at these slow heart rates were driven by abnormal Ca^2+^ handling and the development of CaT alternans, and that changes in CaT alternans threshold resulted from steepening of the SR Ca^2+^ release slope, decreased SR Ca^2+^ uptake efficiency, and decreased inactivation of the RyR. These findings provide important insight into the mechanisms underlying proarrhythmic APD alternans occurring at slow heart rates in cAF patients. Such insight may aid in the development of targeted therapies and new treatment strategies for AF in the future.

## Methods

### Human AF tissue model

In order to investigate ionic mechanisms in human AF that contribute to the generation of atrial alternans at the tissue level, we created a computer model of human atrial tissue incorporating ionic remodeling associated with cAF. The atrial tissue preparation had dimensions of 0.33×0.33×9.9 mm^3^ (Fig. 1A), similar to the one used by Krummen *et al.* [65] Human atrial cell membrane kinetics were represented by a modified version of the Grandi-Pandit-Voigt (GPV) human atrial action potential model [19], which we refer to as the GPVm model. Detailed explanation and justification of the GPVm model modifications are provided in the supplement (S1, S2 Texts). Different types of human atrial tissue were modeled individually as homogenous tissue preparations, with each incorporating ionic changes appropriate for each tissue type. Both control and cAF-remodeled tissue, as well as left and right atrial tissue, were modeled using the parameter changes specified by Grandi *et al.* [19] (see S1 Text). The isotropic bulk conductivity value for the tissue was tuned to produce a conduction velocity of 0.62 m/s in control tissue [59], [66]. When cAF ionic remodeling was incorporated, the same bulk conductivity value produced a conduction velocity of 0.59 m/s. These values are within the reported ranges for control and AF conduction velocities [67].

### Protocols for evaluating alternans in the human AF tissue model

We assessed alternans in the human AF tissue model by applying the clinical pacing protocol used by Narayan *et al.* to induce alternans in AF patients [8]. The tissue model was first initialized at all nodes with steady-state values from a single cell paced at 750-ms CL. The tissue was then paced from the stimulus electrode (Fig. 1A) for 20 beats at 750-ms CL and then for 74 beats at each subsequent CL, starting from 500 ms and shortened in 50-ms steps to 300 ms, and then shortened in 10-ms steps, until loss of capture or conduction block occurred.

Voltage traces from the recording electrode (Fig. 1A) were analyzed for APD alternans. APD was calculated as the time from maximal upstroke velocity to 90% repolarization of V_m_ from phase II amplitude. Alternans magnitude was quantified as the mean magnitude of change in APD over the last 10 pairs of beats (11 beats total). APD alternans normalized magnitude (ANM), obtained by dividing the alternans magnitude by the mean APD over the last 10 beats, was used to compare alternans between cells of varying APD. Alternans onset CL was defined as the longest CL for which ANM was greater than 5% [8].

### Sensitivity of alternans to ionic model parameters

To identify cellular changes which could account for the onset of alternans in AF patients at CLs of 300–500 ms [8], we explored how ANM varied in human AF tissue models of both the left and right atrium as a result of changes in ionic model parameters. Of the 20 ionic model parameters tested, 10 were parameters altered in the GPVm model to represent cAF [19]; others were associated with L-type Ca^2+^ current (I_CaL_), rapidly activating potassium current (I_Kr_), SR uptake, or SR release (Table 1). We scaled parameter values one at a time to 25–200% of the default left or right atrium values specified by Grandi *et al.* [19]; for each parameter value within this range, simulations were conducted to determine the presence of alternans (282 simulations total). In AF patients, average alternans onset CL was>300 ms [8], so pacing and alternans analysis was restricted to CLs≥300 ms.

### Clamping protocols

After identifying conditions under which APD alternans magnitude and onset CL matched clinical observations, we utilized two different clamping approaches in order to investigate the key cellular properties that gave rise to these alternans, as described below. Further explanation of the rationale behind these methods can be found in Results.

#### Ionic model variable clamps

To determine which human atrial ionic model variables drive the occurrence of alternans, we clamped individual ion currents and state variables in a single-cell model paced at a CL exhibiting alternans [15]. A model variable was clamped to its steady-state even or odd beat trace for the duration of 50 beats. This procedure was repeated for different model variables (membrane currents, SR fluxes, and all state variables excluding buffer concentrations), and APD alternans magnitude was quantified at the end of the 50 clamped beats. Additionally, the magnitude of alternans in Δ[Ca^2+^]_i_ was quantified in the same manner as APD alternans magnitude, with Δ[Ca^2+^]_i_ calculated as the difference between peak [Ca^2+^]_i_ during the beat and minimum [Ca^2+^]_i_ during the preceding diastolic interval (DI). Model variables were considered critical for alternans if clamping them to either the even or odd beat reduced both APD and CaT alternans magnitudes by >99% of baseline [15].

#### AP clamp

To evaluate the Ca^2+^ cycling properties of the human atrial cell model under different pacing rates and parameter values, the following equation was used to clamp V_m_ to a generic atrial AP-like waveform so that comparisons between different conditions would not be influenced by variations in V_m_:
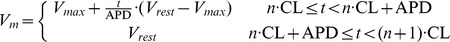
This approach has been used previously to investigate Ca^2+^ cycling properties in ventricular myocyte models [22], [50]. We set V_max_ = 10 mV, V_rest_ = −75 mV, and APD = 200 ms. CL ranged from 200 to 700 ms.

The AP clamp enabled evaluation of Ca^2+^ cycling stability in the human atrial cell model via an iterated map analysis [22], [28], [68]. We used a similar approach as Qu *et al.* [29], where SR load and total Ca^2+^ content of the cell are tracked from beat to beat. In our analysis, Ca^2+^ cycling stability depended upon three iterated map parameters: SR Ca^2+^ release slope (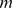), SR Ca^2+^ uptake factor (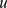), and cellular Ca^2+^ efflux factor (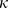). A detailed derivation of the iterated map stability criteria can be found in S1 Text.

To compute the iterated map parameters, a single atrial cell was repeatedly clamped to the AP waveform until model variables reached steady state. Following this, [Ca^2+^]_SR_ was perturbed by ±1% at the beginning of an even beat, and total SR load, release, uptake, and cellular Ca^2+^ efflux per beat were recorded for the following 10 beats. For the Sato-Bers model, the first beat was excluded since it deviated noticeably from the linear response of later beats. This procedure was repeated starting with an odd beat so that data from a total of 40 beats were recorded (36 beats for the Sato-Bers model). Lastly, 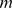, 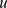, and 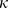 were computed as the slopes of the linear least-squares fit of the data (see S1 Text).

### Numerical methods

The monodomain and ionic model equations were solved using the Cardiac Arrhythmia Research Package (CARP; Cardiosolv, LLC) [69]. Details on the numerical techniques used by CARP have been described previously [70], [71]. A time step of 20 µs was used for all simulations.

## Supporting Information

S1 Figure **Comparison of original and modified versions of the GPV ionic model in tissue.** At 400-ms CL, the original GPV model did not propagate robustly in tissue (black line). When the fast sodium current kinetics was replaced with the kinetics from the Luo-Rudy dynamic model (LRd), normal propagation occurred (blue line). Applying the fast equilibrium approximation to select buffers (see S2 Text) had a negligible effect on simulation results (dotted green line).
(TIF)

S2 Figure **Sensitivity of APD alternans magnitude to ionic model parameters in RA cAF tissue during pacing.** Parameter sensitivity analysis was performed in tissue with the right atrium version of the GPVm model incorporating cAF remodeling, in order to identify ionic model parameters that influence alternans. APD alternans normalized magnitude (ANM) is indicated by the colorbar (>0.05 considered significant). Parameters were scaled one at a time between 25% (short ticks) and 200% (long ticks) of their AF model values (25% increments). Results were similar to those obtained with the left atrium version of the model (see Fig. 2A), with alternans occurring at the longest CLs only when the RyR inactivation rate constant (ki_Ca_) was decreased.
(TIF)

S3 Figure **APD alternans magnitudes in cAF_alt_ tissue.** The tissue preparation was paced from the stimulus electrode (see Fig. 1A), and APD alternans normalized magnitudes (ANMs) were quantified at each cycle length for every node along the tissue. When significant alternans was present in the tissue (ANM>0.05), all nodes had concordant alternans of similar magnitude.
(TIF)

S4 Figure **Voltage and Ca^2+^ odd beat clamps for the single-cell cAF_alt_ model.** Traces of transmembrane potential (V_m_, row 1), intracellular Ca^2+^ ([Ca^2+^]_i_, row 2), and SR Ca^2+^ ([Ca^2+^]_SR_, row 3) from two consecutive beats are superimposed to show alternans between even (red) and odd (blue) beats. Column 1: the unclamped cAF_alt_ cell paced to steady state at 400-ms CL displayed alternans in V_m_ and Ca^2+^. The blue traces depicted in column 1 were used to clamp V_m_ (column 2), [Ca^2+^]_i_ (column 3), or [Ca^2+^]_SR_ (column 4). Alternans persisted when V_m_ or [Ca^2+^]_i_ was clamped, but clamping [Ca^2+^]_SR_ eliminated alternans.
(TIF)

S5 Figure **SR Ca^2+^ release parameter even beat clamps for the single-cell cAF_alt_ model.** Traces of transmembrane potential (V_m_, row 1), intracellular Ca^2+^ ([Ca^2+^]_i_, row 2), and SR Ca^2+^ ([Ca^2+^]_SR_, row 3) from two consecutive beats are superimposed to show alternans between even (red) and odd (blue) beats. Traces from the even beat at 400-ms CL pacing were used to clamp the relevant variable and are shown in row 4. Clamping RyR inactivated probability (RyR_i_, column 1), RyR open probability (RyR_o_, column 2), junctional Ca^2+^ ([Ca^2+^]_j_, column 3), or SR Ca^2+^ release flux (J_SRCarel_, column 4) eliminated alternans in V_m_ and Ca^2+^.
(TIF)

S6 Figure **SR Ca^2+^ release parameter odd beat clamps for the single-cell cAF_alt_ model.** Traces of transmembrane potential (V_m_, row 1), intracellular Ca^2+^ ([Ca^2+^]_i_, row 2), and SR Ca^2+^ ([Ca^2+^]_SR_, row 3) from two consecutive beats are superimposed to show alternans between even (red) and odd (blue) beats. Traces from the odd beat at 400-ms CL pacing were used to clamp the relevant variable and are shown in row 4. Clamping RyR inactivated probability (RyR_i_, column 1), RyR open probability (RyR_o_, column 2), junctional Ca^2+^ ([Ca^2+^]_j_, column 3), or SR Ca^2+^ release flux (J_SRCarel_, column 4) eliminated alternans in V_m_ and Ca^2+^.
(TIF)

S7 Figure **Sub-sarcolemmal parameter clamps for the single-cell cAF_alt_ model.** Traces of transmembrane potential (V_m_, row 1), intracellular Ca^2+^ ([Ca^2+^]_i_, row 2), and SR Ca^2+^ ([Ca^2+^]_SR_, row 3) from two consecutive beats are superimposed to show alternans between even (red) and odd (blue) beats. Traces from the even or odd beat at 400-ms CL pacing were used to clamp the relevant variable and are shown in row 4. Clamping sub-sarcolemmal Ca^2+^ ([Ca^2+^]_sl_) to the even beat (column 1) eliminated alternans in V_m_ and Ca^2+^, but clamping [Ca^2+^]_sl_ to the odd beat (column 2) produced small alternans in V_m_ and [Ca_2+_]_i_ and large alternans in [Ca^2+^]_SR_. Clamping sub-sarcolemmal Na^+^/Ca^2+^ exchanger current (I_NCXsl_) to the even beat (column 3) eliminated alternans in APD but produced large alternans in [Ca^2+^]_i_ and [Ca^2+^]_SR_. Clamping I_NCXsl_ to the odd beat (column 4) eliminated alternans in V_m_ and Ca^2+^.
(TIF)

S8 Figure **Multivariable regression between ionic model parameters and alternans threshold CL.** (A) Bar graph of regression coefficient magnitudes. Twenty ionic model parameters were varied stochastically over 500 simulations to assess their effects on alternans cycle length (CL). Of the 500 simulations, 83 were excluded from the analysis because alternans threshold CL was below 100 ms or above 750 ms. Linear regression coefficients for each of the parameters are plotted in order of decreasing magnitude, with positive values plotted in red and negative values plotted in blue. Asterisks indicate *p*<0.05 for the *t*-statistic. (B) Bar graph of the predicted contribution of parameters to alternans threshold CL in the cAF-remodeled cell. Ten of the twenty parameters used in the regression analysis were altered from control values to represent cAF remodeling (increases and decreases indicated by upward and downward arrows, respectively). Parameters whose changes were predicted to increase (decrease) the alternans CL are plotted in red (blue). Some unaltered parameters had nonzero predicted contributions to alternans threshold CL due to nonzero sample means from the regression analysis. The alternans threshold CL predicted by regression analysis (245 ms) was very close to the actual alternans threshold CL determined by simulation (244 ms).
(TIF)

S9 Figure **Single-cell APD restitution in control model.** With default model parameter values, APD alternans occurred at 200 ms CL (black). When the RyR inactivation rate constant (ki_Ca_) was reduced to 95%, alternans occurred at slightly longer CLs (red). These results were comparable to alternans onset data from control patients [8].
(TIF)

S10 Figure **APD and CaT oscillations in single-cell and tissue models with Sato-Bers RyR formulation.** Control (black), cAF (red), and cAF_alt_ (dotted red line) versions of the model using the Sato-Bers RyR [27] were implemented in single cell (A and B) and in tissue (C and D). In the cAF_alt_ model, the calsequestrin-bound RyR closing rate (k_34_) was decreased by 50%. APD (A and C) and CaT (B and D) restitution data are plotted showing the mean±SD range (control, gray shading, not visible; cAF, pink shading; cAF_alt_, red hatching). Oscillations in APD and CaT included but were not limited to alternans. Oscillations exhibited the reverse of the rate dependence observed in models using the original RyR formulation, with larger oscillations at longer CL. APD oscillations in these models were diminished as compared to the original models (see Fig. 1), and both APD and CaT oscillations were attenuated in tissue.
(TIF)

S1 Text **Supplemental methods.**
(PDF)

S2 Text **Supplemental equations.**
(PDF)
