# Supplementary figures and images for: Disrupted Calcium Release as a Mechanism for Atrial Alternans Associated with Human Atrial Fibrillation
Source: PLoS Comput Biol. 2014 Dec 11;10(12):e1004011. doi: 10.1371/journal.pcbi.1004011 (PMC4263367; doi:10.1371/journal.pcbi.1004011)

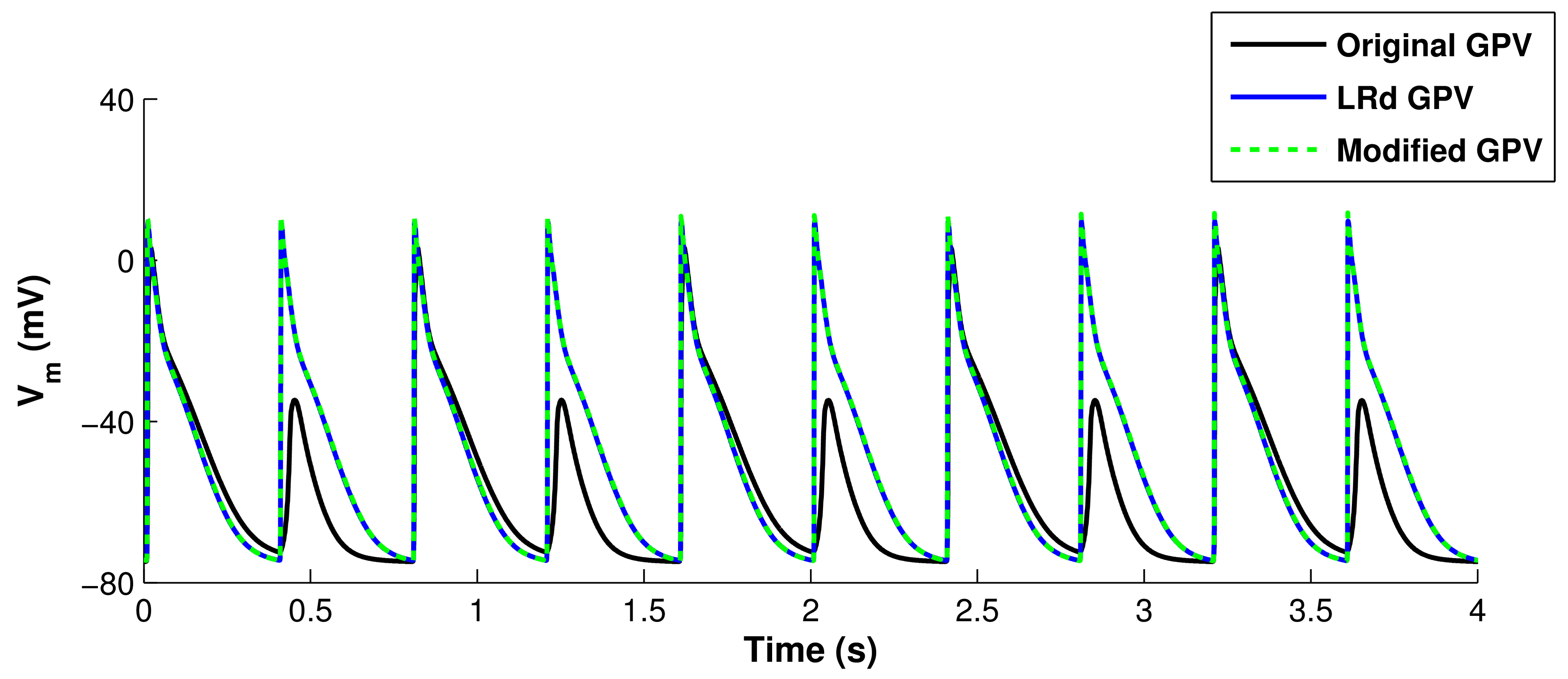

Supplement: S1 Figure — Comparison of original and modified versions of the GPV ionic model in tissue. At 400-ms CL, the original GPV model did not propagate robustly in tissue (black line). When the fast sodium current kinetics was replaced with the kinetics from the Luo-Rudy dynamic model (LRd), normal propagation occurred (blue line). Applying the fast equilibrium approximation to select buffers (see S2 Text) had a negligible effect on simulation results (dotted green line). (TIF) [file pcbi.1004011.s001.tif]

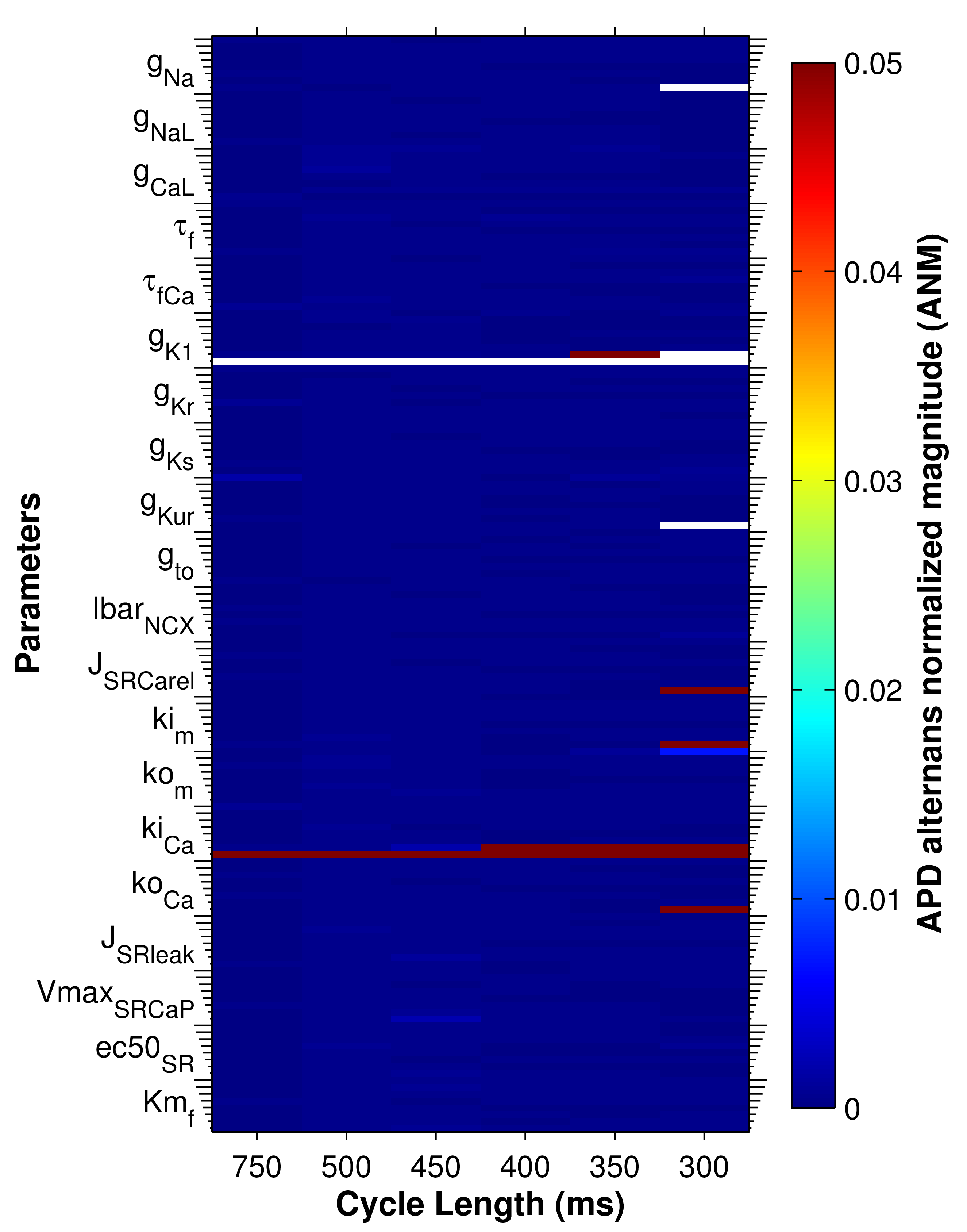

Supplement: S2 Figure — Sensitivity of APD alternans magnitude to ionic model parameters in RA cAF tissue during pacing. Parameter sensitivity analysis was performed in tissue with the right atrium version of the GPVm model incorporating cAF remodeling, in order to identify ionic model parameters that influence alternans. APD alternans normalized magnitude (ANM) is indicated by the colorbar (>0.05 considered significant). Parameters were scaled one at a time between 25% (short ticks) and 200% (long ticks) of their AF model values (25% increments). Results were similar to those obtained with the left atrium version of the model (see Fig. 2A), with alternans occurring at the longest CLs only when the RyR inactivation rate constant (kiCa) was decreased. (TIF) [file pcbi.1004011.s002.tif]

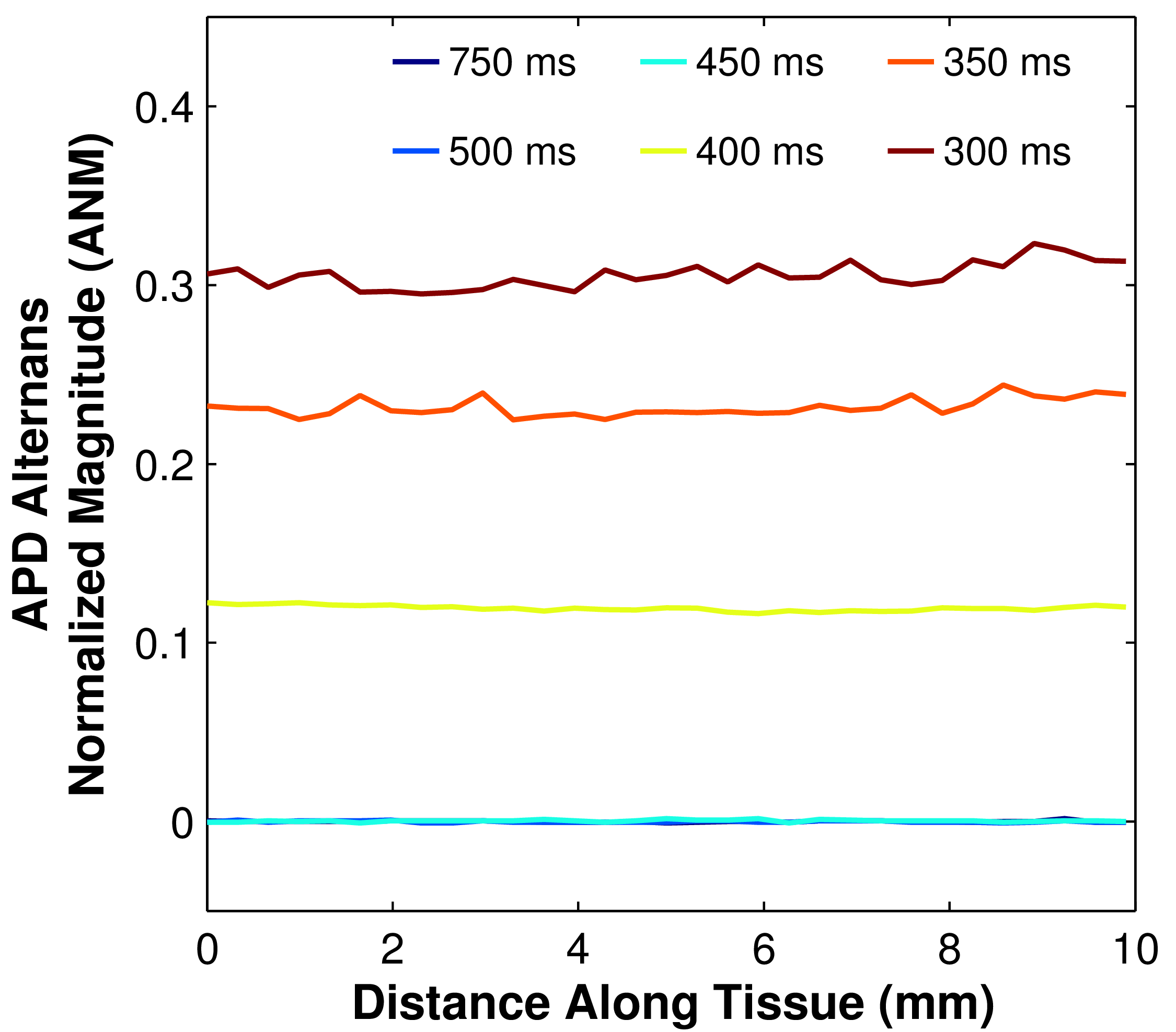

Supplement: S3 Figure — APD alternans magnitudes in cAFalt tissue. The tissue preparation was paced from the stimulus electrode (see Fig. 1A), and APD alternans normalized magnitudes (ANMs) were quantified at each cycle length for every node along the tissue. When significant alternans was present in the tissue (ANM>0.05), all nodes had concordant alternans of similar magnitude. (TIF) [file pcbi.1004011.s003.tif]

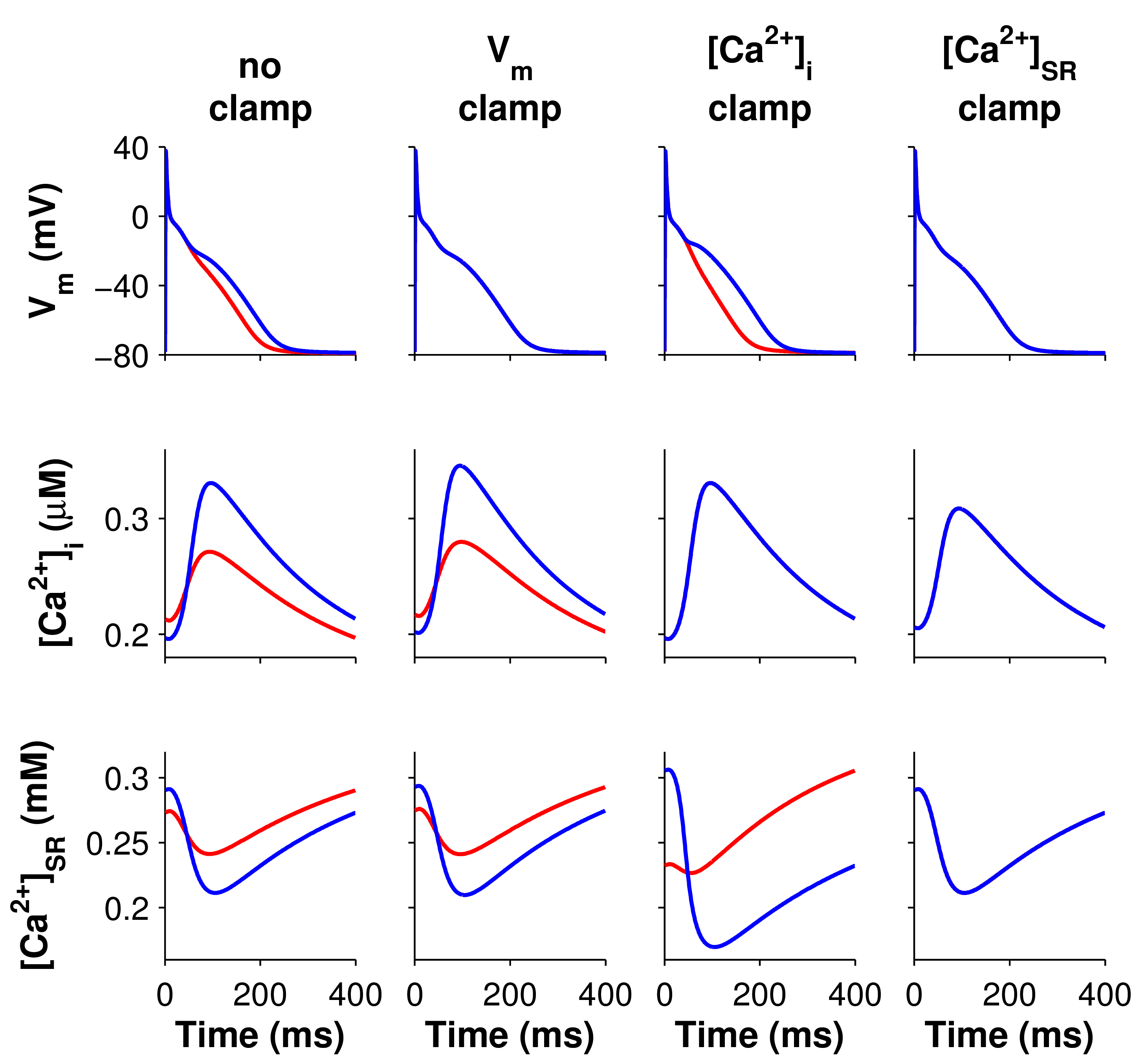

Supplement: S4 Figure — Voltage and Ca2+ odd beat clamps for the single-cell cAFalt model. Traces of transmembrane potential (Vm, row 1), intracellular Ca2+ ([Ca2+]i, row 2), and SR Ca2+ ([Ca2+]SR, row 3) from two consecutive beats are superimposed to show alternans between even (red) and odd (blue) beats. Column 1: the unclamped cAFalt cell paced to steady state at 400-ms CL displayed alternans in Vm and Ca2+. The blue traces depicted in column 1 were used to clamp Vm (column 2), [Ca2+]i (column 3), or [Ca2+]SR (column 4). Alternans persisted when Vm or [Ca2+]i was clamped, but clamping [Ca2+]SR eliminated alternans. (TIF) [file pcbi.1004011.s004.tif]

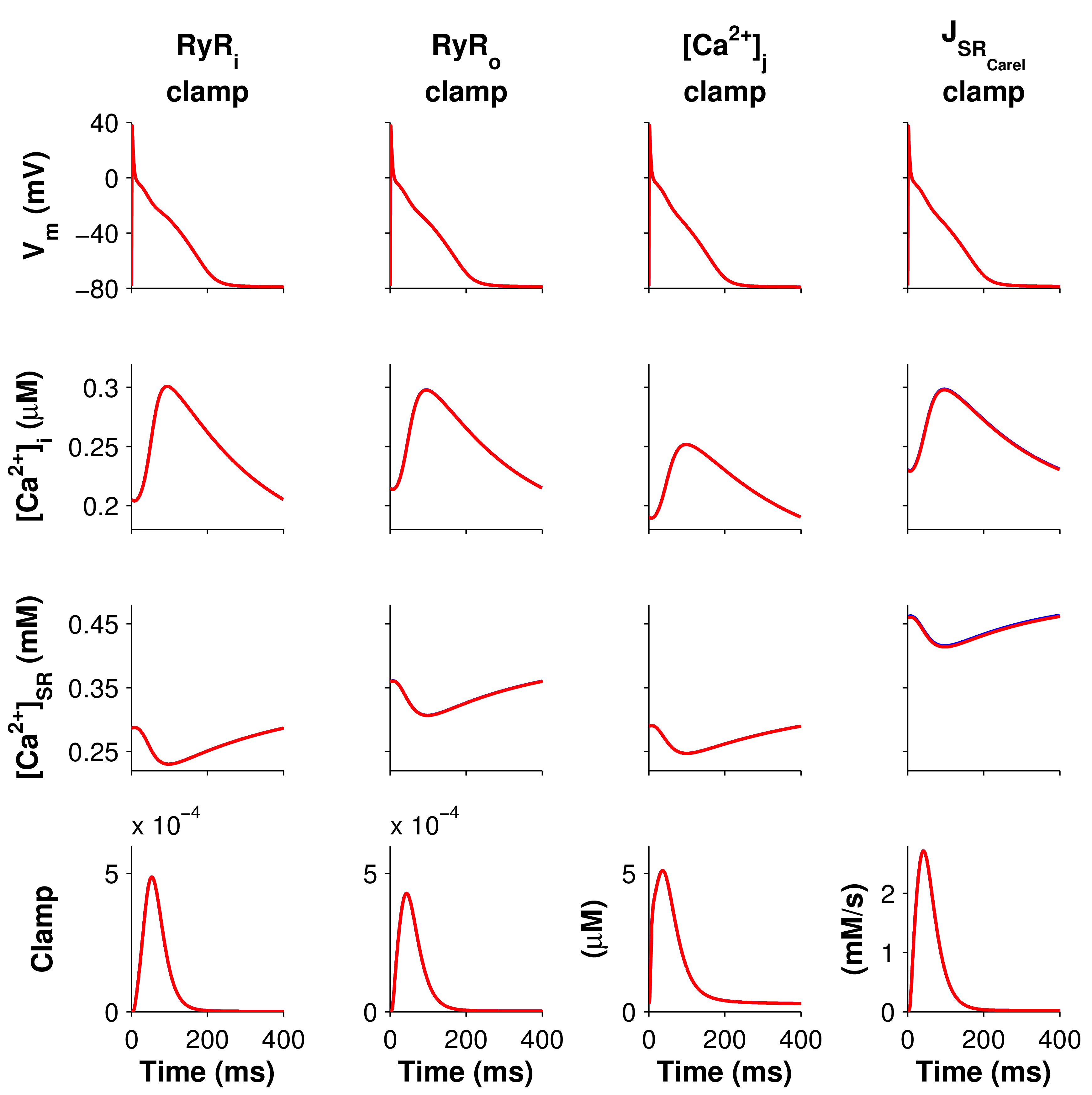

Supplement: S5 Figure — SR Ca2+ release parameter even beat clamps for the single-cell cAFalt model. Traces of transmembrane potential (Vm, row 1), intracellular Ca2+ ([Ca2+]i, row 2), and SR Ca2+ ([Ca2+]SR, row 3) from two consecutive beats are superimposed to show alternans between even (red) and odd (blue) beats. Traces from the even beat at 400-ms CL pacing were used to clamp the relevant variable and are shown in row 4. Clamping RyR inactivated probability (RyRi, column 1), RyR open probability (RyRo, column 2), junctional Ca2+ ([Ca2+]j, column 3), or SR Ca2+ release flux (JSRCarel, column 4) eliminated alternans in Vm and Ca2+. (TIF) [file pcbi.1004011.s005.tif]

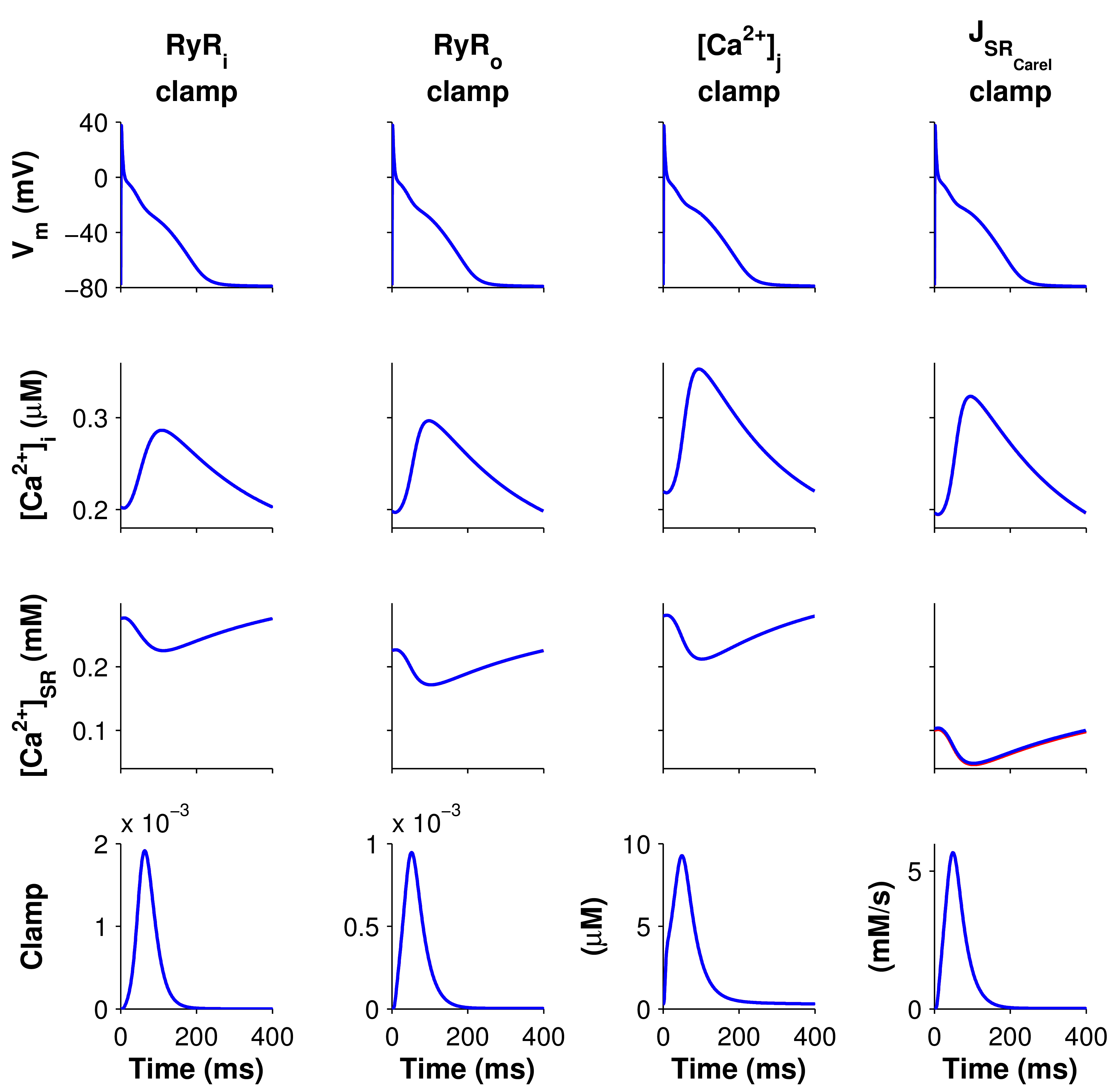

Supplement: S6 Figure — SR Ca2+ release parameter odd beat clamps for the single-cell cAFalt model. Traces of transmembrane potential (Vm, row 1), intracellular Ca2+ ([Ca2+]i, row 2), and SR Ca2+ ([Ca2+]SR, row 3) from two consecutive beats are superimposed to show alternans between even (red) and odd (blue) beats. Traces from the odd beat at 400-ms CL pacing were used to clamp the relevant variable and are shown in row 4. Clamping RyR inactivated probability (RyRi, column 1), RyR open probability (RyRo, column 2), junctional Ca2+ ([Ca2+]j, column 3), or SR Ca2+ release flux (JSRCarel, column 4) eliminated alternans in Vm and Ca2+. (TIF) [file pcbi.1004011.s006.tif]

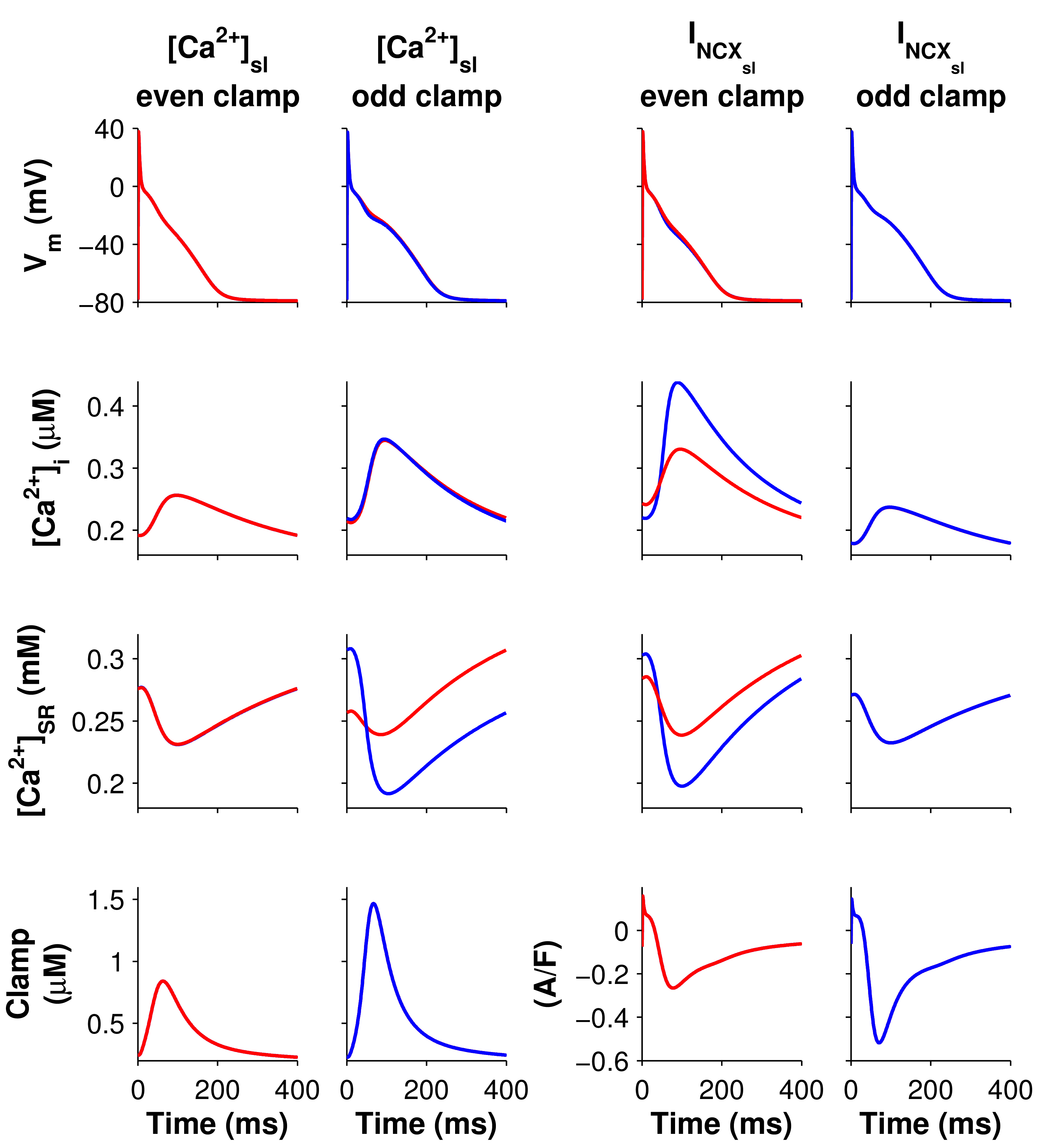

Supplement: S7 Figure — Sub-sarcolemmal parameter clamps for the single-cell cAFalt model. Traces of transmembrane potential (Vm, row 1), intracellular Ca2+ ([Ca2+]i, row 2), and SR Ca2+ ([Ca2+]SR, row 3) from two consecutive beats are superimposed to show alternans between even (red) and odd (blue) beats. Traces from the even or odd beat at 400-ms CL pacing were used to clamp the relevant variable and are shown in row 4. Clamping sub-sarcolemmal Ca2+ ([Ca2+]sl) to the even beat (column 1) eliminated alternans in Vm and Ca2+, but clamping [Ca2+]sl to the odd beat (column 2) produced small alternans in Vm and [Ca2+]i and large alternans in [Ca2+]SR. Clamping sub-sarcolemmal Na+/Ca2+ exchanger current (INCXsl) to the even beat (column 3) eliminated alternans in APD but produced large alternans in [Ca2+]i and [Ca2+]SR. Clamping INCXsl to the odd beat (column 4) eliminated alternans in Vm and Ca2+. (TIF) [file pcbi.1004011.s007.tif]

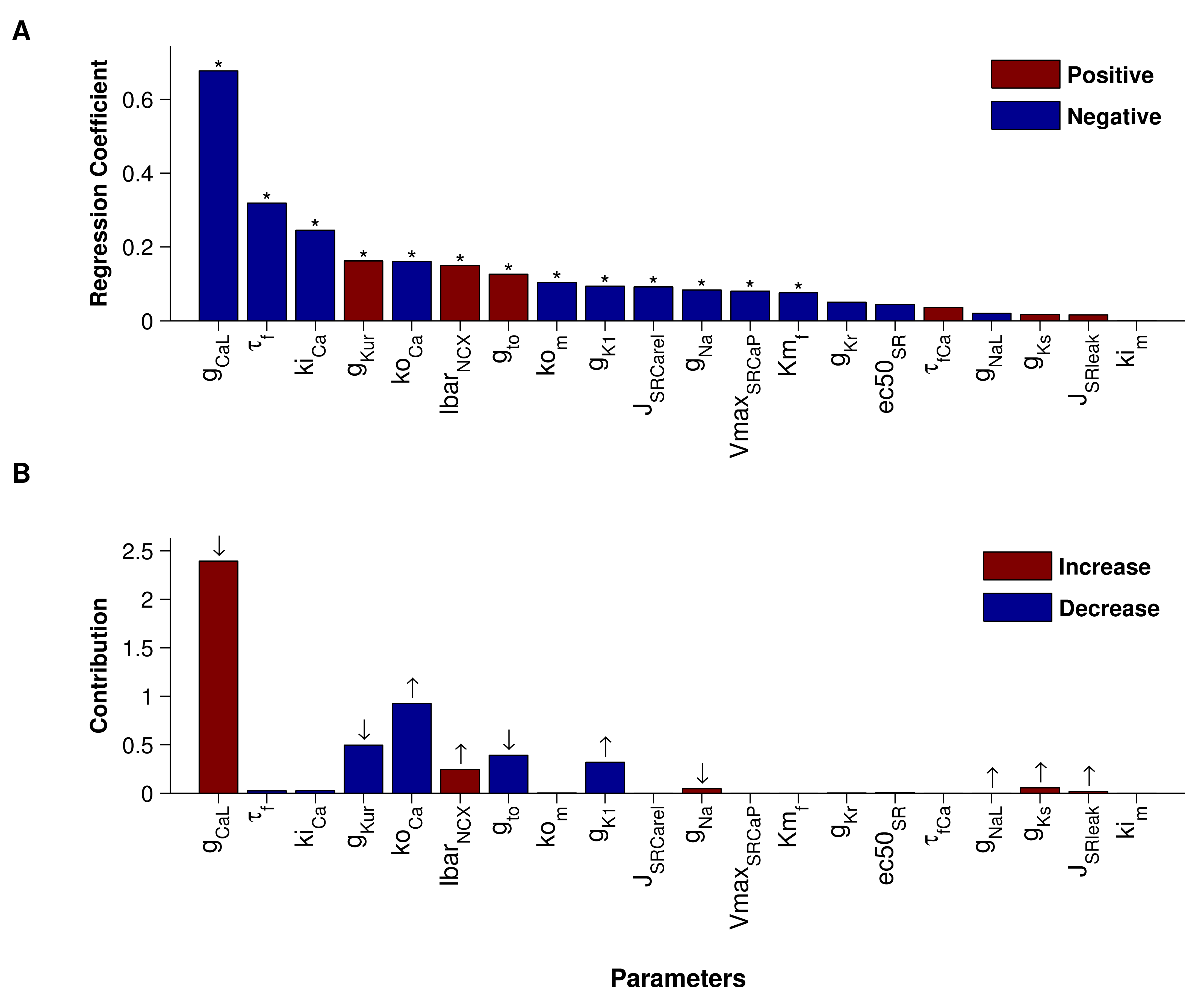

Supplement: S8 Figure — Multivariable regression between ionic model parameters and alternans threshold CL. (A) Bar graph of regression coefficient magnitudes. Twenty ionic model parameters were varied stochastically over 500 simulations to assess their effects on alternans cycle length (CL). Of the 500 simulations, 83 were excluded from the analysis because alternans threshold CL was below 100 ms or above 750 ms. Linear regression coefficients for each of the parameters are plotted in order of decreasing magnitude, with positive values plotted in red and negative values plotted in blue. Asterisks indicate p<0.05 for the t-statistic. (B) Bar graph of the predicted contribution of parameters to alternans threshold CL in the cAF-remodeled cell. Ten of the twenty parameters used in the regression analysis were altered from control values to represent cAF remodeling (increases and decreases indicated by upward and downward arrows, respectively). Parameters whose changes were predicted to increase (decrease) the alternans CL are plotted in red (blue). Some unaltered parameters had nonzero predicted contributions to alternans threshold CL due to nonzero sample means from the regression analysis. The alternans threshold CL predicted by regression analysis (245 ms) was very close to the actual alternans threshold CL determined by simulation (244 ms). (TIF) [file pcbi.1004011.s008.tif]

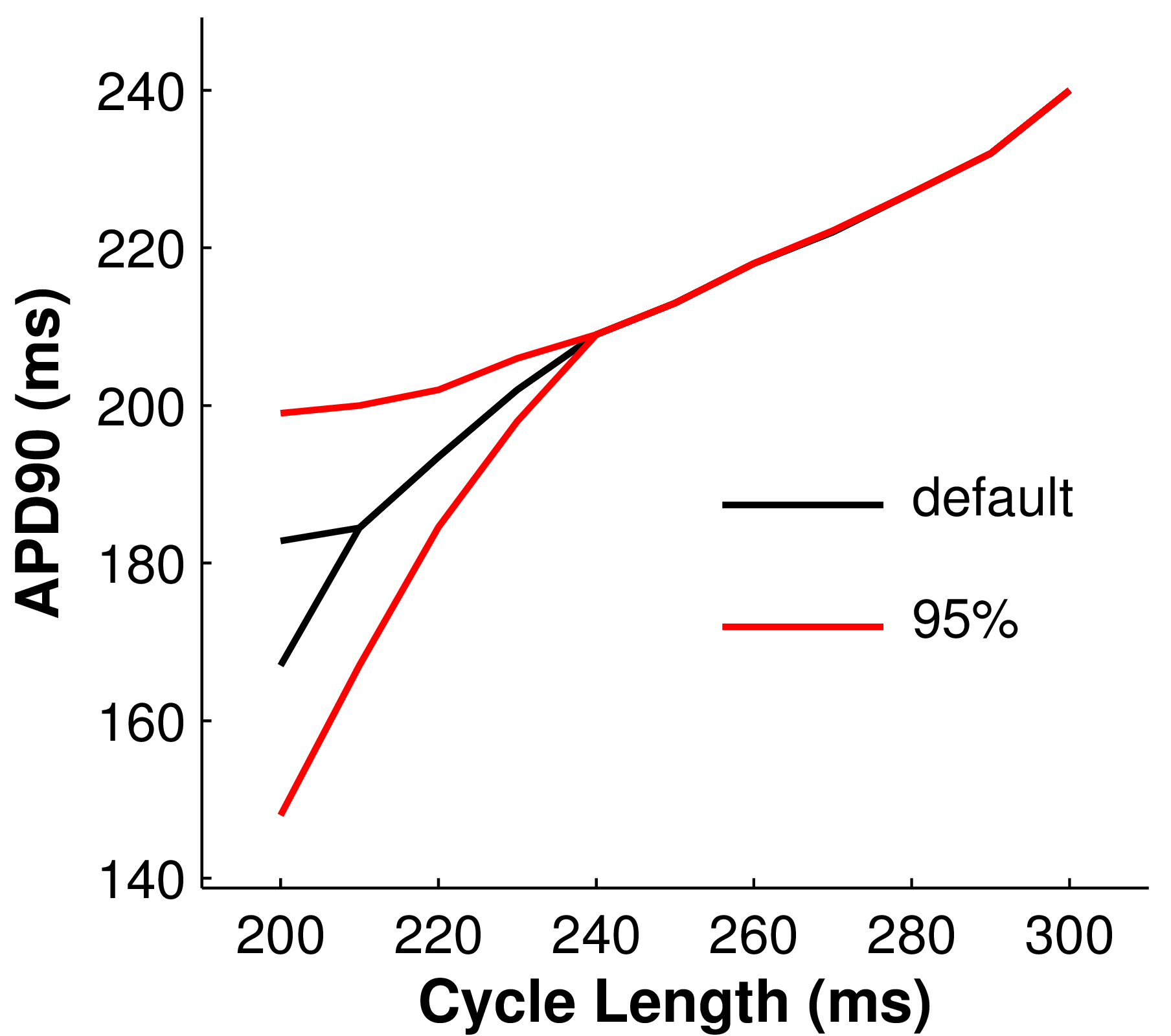

Supplement: S9 Figure — Single-cell APD restitution in control model. With default model parameter values, APD alternans occurred at 200 ms CL (black). When the RyR inactivation rate constant (kiCa) was reduced to 95%, alternans occurred at slightly longer CLs (red). These results were comparable to alternans onset data from control patients [8]. (TIF) [file pcbi.1004011.s009.tif]

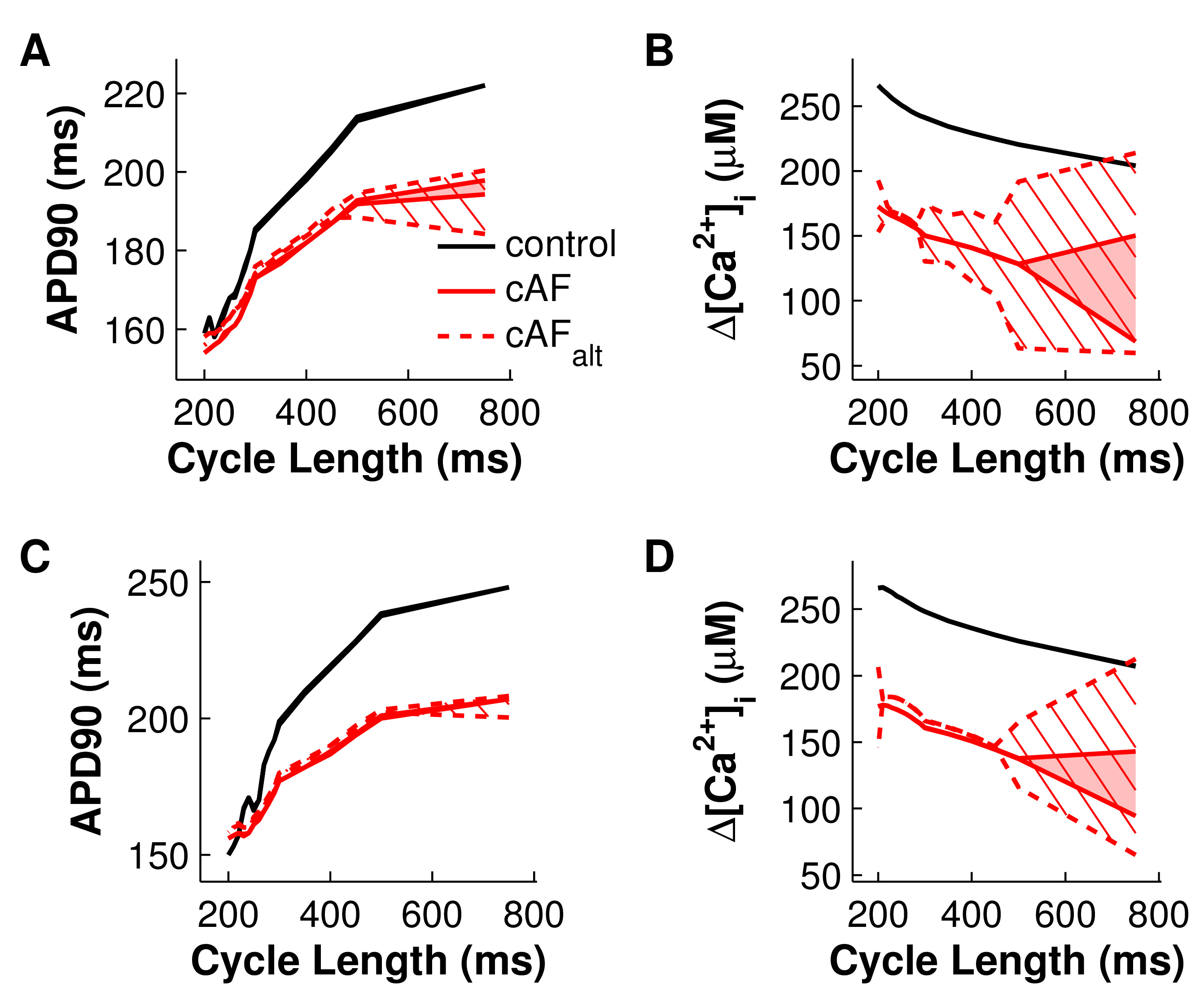

Supplement: S10 Figure — APD and CaT oscillations in single-cell and tissue models with Sato-Bers RyR formulation. Control (black), cAF (red), and cAFalt (dotted red line) versions of the model using the Sato-Bers RyR [27] were implemented in single cell (A and B) and in tissue (C and D). In the cAFalt model, the calsequestrin-bound RyR closing rate (k34) was decreased by 50%. APD (A and C) and CaT (B and D) restitution data are plotted showing the mean±SD range (control, gray shading, not visible; cAF, pink shading; cAFalt, red hatching). Oscillations in APD and CaT included but were not limited to alternans. Oscillations exhibited the reverse of the rate dependence observed in models using the original RyR formulation, with larger oscillations at longer CL. APD oscillations in these models were diminished as compared to the original models (see Fig. 1), and both APD and CaT oscillations were attenuated in tissue. (TIF) [file pcbi.1004011.s010.tif]
