# Supplementary material for: Disrupted Calcium Release as a Mechanism for Atrial Alternans Associated with Human Atrial Fibrillation
Source: PLoS Comput Biol. 2014 Dec 11;10(12):e1004011. doi: 10.1371/journal.pcbi.1004011 (PMC4263367; doi:10.1371/journal.pcbi.1004011)
Supplement: S1 Text — Supplemental methods. (PDF) [file pcbi.1004011.s011.pdf]

# Supplemental Methods

## Modifications to the GPV model

The Grandi-Pandit-Voigt (GPV) model failed to propagate robustly when paced at faster rates (Fig. S1, black trace). We substituted the fast sodium current ($I_{Na}$) kinetics of the original model with the formulation from the Luo-Rudy dynamic model (LRd)[1] to achieve normal propagation, as done previously[2] (Fig. S1, blue trace). $I_{Na}$ conductance ($g_{Na}$) was set to 14 mS/μF to reproduce maximum upstroke velocity (208 V/s) and action potential amplitude (105 mV) values reported previously[3,4]. In addition, we reduced the number of state variables in the GPV model using a rapid equilibrium approximation for $Ca^{2+}$ and $Na^{+}$ buffers with fast kinetics (Text S2). This decreased the computation time for tissue simulations by about 6X. The modified GPV model (GPVm) with LRd $I_{Na}$ kinetics and fewer state variables was used for all simulations in this study unless otherwise indicated.

## Intra-atrial heterogeneity and cAF remodeling

For the GPVm model, we implemented the changes described in Grandi *et al.* for the left atrium (LA) and right atrium (RA)[5]. In normal cells, the maximum conductance of $I_{Kur}$ ($g_{Kur}$) is increased by 20% in the RA as compared to the LA. In cAF cells, the maximum conductances of $I_{Kur}$ and $I_{to}$ are differentially downregulated from their normal levels, with $g_{Kur}$ decreased by 45% and 55% and $g_{to}$ decreased by 45% and 80% in the LA and RA, respectively. Additionally, modifications of the action potential under conditions of cAF included the following changes that occurred in both LA and RA: a 10% decrease in $g_{Na}$, addition of a late component to the sodium current ($I_{NaL}$), a 50% decrease in $g_{CaL}$, a 40% increase in $Ibar_{NCX}$, a 3-fold increase in the ryanodine receptor (RyR) $Ca^{2+}$-dependent activation rate ($ko_{Ca}$), a 25% increase in sarcoplasmic reticulum (SR) $Ca^{2+}$ leak, a 2-fold increase in $g_{Ks}$, and a 2-fold increase in $g_{K1}$.

## Sato-Bers RyR model implementation

Model parameters which were modified for implementation of the Sato-Bers RyR model[6] are listed in Table 2. The SR was divided into junctional (JSR) and network (NSR) compartments, the former of which contained the $Ca^{2+}$ buffer calsequestrin (CSQN). Equations for CSQN buffering in the Sato-Bers model were derived from Restrepo *et al.*[7]:

$$\beta_{JSR} = \left(1 + \frac{B_{max_{csqn}}\left(K_C n + \frac{\partial n}{\partial Ca_{JSR}} Ca_{JSR}\left(K_c + Ca_{JSR}\right)\right)}{\left(K_c + Ca_{JSR}\right)^2}\right)^{-1} \quad \text{(S1)}$$

$$n = \widehat{M} n_M + \left(1 - \widehat{M}\right) n_D \quad \text{(S2)}$$

$$\widehat{M} = \frac{\left(1 + 8\rho B_{max_{csqn}}\right)^{1/2} - 1}{4\rho B_{max_{csqn}}} \quad \text{(S3)}$$

$$\rho = \frac{\rho_\infty {Ca_{JSR}}^{h_{sr}}}{K^{h_{sr}} + {Ca_{JSR}}^{h_{sr}}} \quad \text{(S4)}$$

The RyR equations were updated as described in Sato and Bers[6]:

$$k_{12} = \frac{K_u {Ca_j}^2}{{K_{cp}}^2 + {Ca_j}^2} + w \quad \text{(S5)}$$

$$k_{43} = \frac{K_b {Ca_j}^2}{{K_{cp}}^2 + {Ca_j}^2} + w \quad \text{(S6)}$$

$$k_{21} = 0.5 \text{ ms}^{-1} \quad \text{(S7)}$$

$$k_{34} = 3.3 \text{ ms}^{-1} \quad \text{(S8)}$$

$$k_{14} = \frac{\widehat{M} {\tau_b}^{-1} B_{max_{csqn}}}{B_{CSQN}^0} \quad \text{(S9)}$$

$$k_{23} = \frac{\widehat{M} {\tau_b}^{-1} B_{max_{csqn}}}{B_{CSQN}^0} \quad \text{(S10)}$$

$$k_{41} = \tau_u{}^{-1} \tag{S11}$$

$$k_{32} = \frac{k_{41}k_{12}k_{23}k_{34}}{k_{43}k_{14}k_{21}} \tag{S12}$$

Equation S12 was modified to satisfy detailed balance.

## Iterated map analysis

We used an iterated map analysis to derive $Ca^{2+}$ cycling stability criteria. For small SR load perturbations near steady state, total SR release ($R_n$) and uptake ($U_n$) on each beat changed linearly from beat to beat[8,9]:

$$\Delta R_n = m\Delta l_n \tag{S13}$$

$$\Delta U_n = u\Delta c^p{}_n \tag{S14}$$

$m$ is the SR $Ca^{2+}$ release slope, $u$ is the SR $Ca^{2+}$ uptake factor, and $\Delta l_n$ and $\Delta c^p{}_n$ are the changes in total SR load and peak cytoplasmic $Ca^{2+}$, respectively, from beats $n-1$ to $n$. We did not consider $Ca^{2+}$-induced $Ca^{2+}$ release (CICR) and SR leak separately since both were linearly dependent on SR load near steady state and their effects added linearly. Peak cytoplasmic $Ca^{2+}$ was defined as:

$$c^p{}_n = b_n - l_n + R_n \tag{S15}$$

where $b_n$ is the total $Ca^{2+}$ content in the cell at the start of beat $n$. Equations S13-S15 were used to construct the first mapping equation describing the change in SR load[8–10]:

$$\Delta l_{n+1} = \Delta l_n - m\Delta l_n + u(\Delta b_n - \Delta l_n + m\Delta l_n) \tag{S16}$$

Note that we did not assume $\Delta b_n$ to be zero. We also incorporated a new equation for net $Ca^{2+}$ efflux from the cell ($E_n$), which depended linearly on peak cytoplasmic $Ca^{2+}$ for small perturbations near steady-state:

$$\Delta E_n = \kappa\Delta c^p{}_n \tag{S17}$$

$\kappa$ is the sarcolemmal $Ca^{2+}$ efflux factor. Equations S13, S14, and S17 were used to find the linear least squares fit values of $m$, $u$, and $\kappa$ based on $R_n$, $U_n$, $E_n$, $c^p{}_n$, and $l_n$.

The $Ca^{2+}$ efflux term (Eq. S17) was used to construct the second mapping equation describing the change in total $Ca^{2+}$ content[10]:

$$\Delta b_{n+1} = \Delta b_n - \kappa(\Delta b_n - \Delta l_n + m\Delta l_n) \tag{S18}$$

Though iterated map analysis lacking this second mapping equation has been previously used[9], the addition of the second equation provided more accurate theoretical predictions of $Ca^{2+}$ alternans thresholds in our simulations.

From Eq. S16 and S18, we obtained the following Jacobian matrix for the system[7,10]:

$$\begin{bmatrix} (1-m)(1-u) & u \\ \kappa(1-m) & 1-\kappa \end{bmatrix} \tag{S19}$$

The criteria for stability are that both eigenvalues of the Jacobian have absolute value less than 1:

$$\frac{1}{2}\left|(1-m)(1-u) - \kappa + 1 \pm \sqrt{((1-m)(1-u) - (1-\kappa))^2 + 4u\kappa(1-m)}\right| < 1 \tag{S20}$$

Under physiological conditions where the iterated map parameters are positive and $u$ and $\kappa$ are less than 1, the iterated map parameters must satisfy

$$m < \frac{\kappa - 2}{2u + \kappa - 2} + 1, \qquad 2\mathrm{u} + \kappa < 2 \tag{S21}$$

and

$$m < \frac{1}{u + \kappa - 1} + 1, \qquad \mathrm{u} + \kappa > 1 \tag{S22}$$

for $Ca^{2+}$ cycling to be stable.

## Regression analysis

We used multivariable regression analysis methods from Sobie *et al.* to estimate the contribution of model parameters to the alternans threshold pacing cycle length (CL)[11]. Twenty model parameters (Table 1) were scaled (from control LA values) stochastically and independently according to a lognormal distribution with a

median of 1 and $\sigma = 0.2$. A total of 500 sets of parameter scaling values were generated and used in single-cell simulations.

To determine the alternans threshold CL (output) for a given set of parameter scaling values (input), each cell was first paced to steady state at a CL of 400 ms. Then CL was progressively increased or decreased by 1 ms every 100 beats until APD alternans ceased (alternans $\leq 1\%$) or began (alternans $> 1\%$), depending on whether alternans was present at a CL of 400 ms or not. Alternans threshold CL was defined as the shortest CL at which alternans did not occur. Any cell in which alternans persisted at CLs up to 750 ms or in which alternans was absent at CLs down to 100 ms was excluded from the analysis. Input and output matrices were log-transformed, then mean-centered and normalized by standard deviations (column-wise), before performing linear regression[11]. The regression coefficients obtained by this method indicate which parameters the model is most sensitive to with regards to alternans threshold CL, under assumptions of linearity. Linear regression was performed using MATLAB's LinearModel.fit function. Each parameter coefficient was considered significant if the $p$-value of its $t$-statistic was greater than 0.05. To evaluate the predictive ability of the regression analysis, we multiplied the regression coefficients by parameter scaling values for the cAF model (log-transformed, mean-centered, and normalized) to obtain the predicted contribution of each parameter to changes in alternans CL[12].

## References


1. Luo CH, Rudy Y (1994) A dynamic model of the cardiac ventricular action potential. I. Simulations of ionic currents and concentration changes. Circ Res 74: 1071–1096.

2. Deo M, Ruan Y, Pandit S V, Shah K, Berenfeld O, et al. (2013) KCNJ2 mutation in short QT syndrome 3 results in atrial fibrillation and ventricular proarrhythmia. Proc Natl Acad Sci U S A 110: 4291–4296.

3. Workman AJ, Kane KA, Rankin AC (2001) The contribution of ionic currents to changes in refractoriness of human atrial myocytes associated with chronic atrial fibrillation. Cardiovasc Res 52: 226–235.

4. Courtemanche M, Ramirez RJ, Nattel S (1998) Ionic mechanisms underlying human atrial action potential properties: insights from a mathematical model. Am J Physiol Hear Circ Physiol 275: H301–321.

5. Grandi E, Pandit S V, Voigt N, Workman AJ, Dobrev D, et al. (2011) Human Atrial Action Potential and Ca2+ Model: Sinus Rhythm and Chronic Atrial Fibrillation. Circ Res 109: 1055–1066.

6. Sato D, Bers DM (2011) How does stochastic ryanodine receptor-mediated Ca leak fail to initiate a Ca spark? Biophys J 101: 2370–2379.

7. Restrepo JG, Weiss JN, Karma A (2008) Calsequestrin-mediated mechanism for cellular calcium transient alternans. Biophys J 95: 3767–3789.

8. Weiss JN, Karma A, Shiferaw Y, Chen P-S, Garfinkel A, et al. (2006) From pulsus to pulseless: the saga of cardiac alternans. Circ Res 98: 1244–1253.

9. Xie L-H, Sato D, Garfinkel A, Qu Z, Weiss JN (2008) Intracellular Ca alternans: coordinated regulation by sarcoplasmic reticulum release, uptake, and leak. Biophys J 95: 3100–3110.

10. Qu Z, Shiferaw Y, Weiss J (2007) Nonlinear dynamics of cardiac excitation-contraction coupling: An iterated map study. Phys Rev E 75: 011927.

11. Sobie EA (2009) Parameter sensitivity analysis in electrophysiological models using multivariable regression. Biophys J 96: 1264–1274.

12. Sarkar AX, Christini DJ, Sobie EA (2012) Exploiting mathematical models to illuminate electrophysiological variability between individuals. J Physiol 590: 2555–2567.
