# Supplementary material for: Disrupted Calcium Release as a Mechanism for Atrial Alternans Associated with Human Atrial Fibrillation
Source: PLoS Comput Biol. 2014 Dec 11;10(12):e1004011. doi: 10.1371/journal.pcbi.1004011 (PMC4263367; doi:10.1371/journal.pcbi.1004011)
Supplement: S2 Text — Supplemental equations. (PDF) [file pcbi.1004011.s012.pdf]

Supplemental Equations

Ionic Compartment	Rapid Buffer(s)	Modified Equations
Ca_i	CaM<br><br>SRB	$J_{CaB_{cytosol}} = \dot{TnC_l} + \dot{TnC_{h_c}} + \dot{Myo_c}$<br><br>$\beta_{Ca_i} = \left(1 + \frac{B_{max_{CaM}} \cdot \frac{k_{off_{CaM}}}{k_{on_{CaM}}}}{\left(\frac{k_{off_{CaM}}}{k_{on_{CaM}}} + Ca_i\right)^2} + \frac{B_{max_{SR}} \cdot \frac{k_{off_{sr}}}{k_{on_{sr}}}}{\left(\frac{k_{off_{sr}}}{k_{on_{sr}}} + Ca_i\right)^2}\right)^{-1}$<br><br>$\dot{Ca_i} = \beta_{Ca_i} \cdot \left(-J_{serca} \cdot \frac{V_{sr}}{V_{myo}} - J_{CaB_{cytosol}} + \frac{J_{Ca_{slmyo}}}{V_{myo}} \cdot (Ca_{sl} - Ca_i)\right)$
Ca_j	SLL_j<br><br>SLH_j	$\beta_{Ca_j} = \left(1 + \frac{B_{max_{SL_{low_j}}} \cdot \frac{k_{off_{sl_l}}}{k_{on_{sl_l}}}}{\left(\frac{k_{off_{sl_l}}}{k_{on_{sl_l}}} + Ca_j\right)^2} + \frac{B_{max_{SL_{high_j}}} \cdot \frac{k_{off_{sl_h}}}{k_{on_{sl_h}}}}{\left(\frac{k_{off_{sl_h}}}{k_{on_{sl_h}}} + Ca_j\right)^2}\right)^{-1}$<br><br>$\dot{Ca_j} = \beta_{Ca_j} \cdot \left(-I_{Ca_{tot_{junc}}} \cdot \frac{C_{mem}}{V_{junc} \cdot 2 \cdot Frdy} + \frac{J_{Ca_{juncsl}}}{V_{junc}} \cdot (Ca_{sl} - Ca_j)\right.$<br><br>$\left. + J_{SR_{Ca_{rel}}} \cdot \frac{V_{sr}}{V_{junc}} + J_{SR_{leak}} \cdot \frac{V_{myo}}{V_{junc}}\right)$
Ca_{sl}	SLL_{sl}<br><br>SLH_{sl}	$\beta_{Ca_{sl}} = \left(1 + \frac{B_{max_{SL_{low_{sl}}}} \cdot \frac{k_{off_{sl_l}}}{k_{on_{sl_l}}}}{\left(\frac{k_{off_{sl_l}}}{k_{on_{sl_l}}} + Ca_{sl}\right)^2} + \frac{B_{max_{SL_{high_{sl}}}} \cdot \frac{k_{off_{sl_h}}}{k_{on_{sl_h}}}}{\left(\frac{k_{off_{sl_h}}}{k_{on_{sl_h}}} + Ca_{sl}\right)^2}\right)^{-1}$<br><br>$\dot{Ca_{sl}} = \beta_{sl} \cdot \left(-I_{Ca_{tot_{sl}}} \cdot \frac{C_{mem}}{V_{sl} \cdot 2 \cdot Frdy} + \frac{J_{Ca_{juncsl}}}{V_{sl}} \cdot (Ca_j - Ca_{sl})\right.$<br><br>$\left. + \frac{J_{Ca_{slmyo}}}{V_{sl}} \cdot (Ca_i - Ca_{sl})\right)$

Ionic Compartment	Rapid Buffer(s)	Modified Equations
Ca_{SR}	$Csqn_b$	$$\beta_{Ca_{SR}} = \left(1 + \frac{B_{max_{csqn}} \cdot \frac{k_{off_{csqn}}}{k_{on_{csqn}}}}{\left(\frac{k_{off_{csqn}}}{k_{on_{csqn}}} + Ca_{SR}\right)^2}\right)^{-1}$$ $$\dot{Ca}_{SR} = \beta_{Ca_{SR}} \cdot \left(J_{serca} - \left(J_{SR_{leak}} \cdot \frac{V_{myo}}{V_{sr}} + J_{SR_{Ca_{rel}}}\right)\right)$$
Na_j	Na_{B_j}	$$\beta_{Na_j} = \left(1 + \frac{B_{max_{Na_j}} \cdot \frac{k_{off_{Na}}}{k_{on_{Na}}}}{\left(\frac{k_{off_{Na}}}{k_{on_{Na}}} + Na_j\right)^2}\right)^{-1}$$ $$\dot{Na}_j = \beta_{Na_j} \cdot \left(-I_{Na_{tot_{junc}}} \cdot \frac{C_{mem}}{V_{junc} \cdot Frdy} + \frac{J_{Na_{juncsl}}}{V_{junc}} \cdot (Na_{sl} - Na_j)\right)$$
Na_{sl}	$Na_{B_{sl}}$	$$\beta_{Na_{sl}} = \left(1 + \frac{B_{max_{Na_{sl}}} \cdot \frac{k_{off_{Na}}}{k_{on_{Na}}}}{\left(\frac{k_{off_{Na}}}{k_{on_{Na}}} + Na_{sl}\right)^2}\right)^{-1}$$ $$\dot{Na}_{sl} = \beta_{Na_{sl}} \cdot \left(-I_{Na_{tot_{sl}}} \cdot \frac{C_{mem}}{V_{sl} \cdot Frdy} + \frac{J_{Na_{juncsl}}}{V_{sl}} \cdot (Na_j - Na_{sl}) + \frac{J_{Na_{slmyo}}}{V_{sl}} \cdot (Na_i - Na_{sl})\right)$$
